# Predicting male fertility from the sperm methylome: application to 120 bulls with hundreds of artificial insemination records

**DOI:** 10.1186/s13148-022-01275-x

**Published:** 2022-04-27

**Authors:** Valentin Costes, Aurélie Chaulot-Talmon, Eli Sellem, Jean-Philippe Perrier, Anne Aubert-Frambourg, Luc Jouneau, Charline Pontlevoy, Chris Hozé, Sébastien Fritz, Mekki Boussaha, Chrystelle Le Danvic, Marie-Pierre Sanchez, Didier Boichard, Laurent Schibler, Hélène Jammes, Florence Jaffrézic, Hélène Kiefer

**Affiliations:** 1 INRAE, BREED, Université Paris-Saclay, UVSQ, 78350 Jouy-en-Josas, France; 2 Ecole Nationale Vétérinaire d’Alfort, BREED, 94700 Maisons-Alfort, France; 3 R&D Department, ALLICE, 149 rue de Bercy, 75012 Paris, France; 4 Université Paris-Saclay, AgroParisTech, INRAE, GABI, 78350 Jouy-en-Josas, France

**Keywords:** Male fertility, DNA methylation, Predictive model, Sperm, Cattle

## Abstract

**Background:**

Conflicting results regarding alterations to sperm DNA methylation in cases of spermatogenesis defects, male infertility and poor developmental outcomes have been reported in humans. Bulls used for artificial insemination represent a relevant model in this field, as the broad dissemination of bull semen considerably alleviates confounding factors and enables the precise assessment of male fertility. This study was therefore designed to assess the potential for sperm DNA methylation to predict bull fertility.

**Results:**

A unique collection of 100 sperm samples was constituted by pooling 2–5 ejaculates per bull from 100 Montbéliarde bulls of comparable ages, assessed as fertile (*n* = 57) or subfertile (*n* = 43) based on non-return rates 56 days after insemination. The DNA methylation profiles of these samples were obtained using reduced representation bisulfite sequencing. After excluding putative sequence polymorphisms, 490 fertility-related differentially methylated cytosines (DMCs) were identified, most of which were hypermethylated in subfertile bulls. Interestingly, 46 genes targeted by DMCs are involved in embryonic and fetal development, sperm function and maturation, or have been related to fertility in genome-wide association studies; five of these were further analyzed by pyrosequencing. In order to evaluate the prognostic value of fertility-related DMCs, the sperm samples were split between training (*n* = 67) and testing (*n* = 33) sets. Using a Random Forest approach, a predictive model was built from the methylation values obtained on the training set. The predictive accuracy of this model was 72% on the testing set and 72% on individual ejaculates collected from an independent cohort of 20 bulls.

**Conclusion:**

This study, conducted on the largest set of bull sperm samples so far examined in epigenetic analyses, demonstrated that the sperm methylome is a valuable source of male fertility biomarkers. The next challenge is to combine these results with other data on the same sperm samples in order to improve the quality of the model and better understand the interplay between DNA methylation and other molecular features in the regulation of fertility. This research may have potential applications in human medicine, where infertility affects the interaction between a male and a female, thus making it difficult to isolate the male factor.

**Supplementary Information:**

The online version contains supplementary material available at 10.1186/s13148-022-01275-x.

## Background

Understanding the causes of infertility and subfertility is a challenge in human medicine as these conditions affect approximately 15% of couples. Infertility is defined as an inability to conceive a child after 12 months of unprotected intercourse. In 30% of cases, male factors are recognized as the primary cause of infertility because anatomical, hormonal or known genetic anomalies have been clearly diagnosed but no female factors have been identified [1–3]. However, in 15–30% of cases, male infertility is described as idiopathic [1, 4], meaning that the aforementioned causes have been excluded. Altered semen parameters are commonly observed in cases of idiopathic male infertility, suggesting that unidentified factors that impair spermatogenesis might have compromised fertility [4]. Idiopathic infertility also affects normozoospermic patients, thus making it more difficult to determine the causes and highlighting the need for additional analyses of sperm in order to deepen our understanding of fertility [5].

Mature spermatozoa represent the ultimate form of male germ cell differentiation, which is a tightly regulated process that starts in utero and is achieved in adulthood during spermatogenesis. Sperm cells are meant to survive outside the organism, fertilize an oocyte and contribute to a new individual; and although they are transcriptionally inactive, they bear a remarkable epigenome in line with their high degree of specialization and unique nature [5]. Thus DNA methylation, chromatin, as well as non-coding RNAs, all display unique features inherited from sperm differentiation that are also essential for embryonic development [6, 7]. It has been postulated that alterations to this sperm-specific epigenome due to lifestyle factors, advanced age, medical conditions or environmental exposure may provide an explanation for male infertility or subfertility [8–13]. Of all epigenetic mechanisms, DNA methylation has been a particular focus for studies on male fertility. Manipulations of DNA methylation in pharmacological or genetic models have indeed demonstrated the essential role it plays in male germ cell differentiation and fertility [14–16]. Alterations to the sperm methylome caused by advanced paternal age or exposure to harmful conditions have also been reported to interfere with male fertility, pregnancy outcomes and the health of the next generation [17–21]. Furthermore, from a technical point of view, the starting point for all DNA methylation assays is the preparation of high quality genomic DNA, which is relatively straightforward when compared to other types of epigenetic assays. Standardized platforms such as the Illumina EPIC assay or earlier versions have thus been developed in humans, opening the way to the analysis of large cohorts at a genome scale [22].

Numerous studies in human cohorts have identified specific DNA methylation profiles associated with altered semen parameters [23–25], embryo quality after in vitro fertilization [26], and infertility or subfertility in normozoospermic patients [27, 28]. However, these studies did not reach a consensus regarding a DNA methylation signature for subfertility or infertility [29], with the exception of specific alterations targeting imprinting genes [30], although this view has also been disputed recently [31]. These inconsistent results may have technical or biological origins, such as the diversity of the methods used to process semen and obtain DNA methylation data or the heterogeneity of phenotypes associated with infertility [29]. They may also be related to confounding factors that are inherent to studies on human fertility which make it very difficult to isolate male factors (one male partner, one female partner, both living in the same environment) [32].

Unlike the situation in humans, the widespread use of artificial insemination (AI) in dairy cattle and the dissemination of bull semen to many herds markedly reduces these limitations; the bull is therefore an excellent animal model to investigate the etiology of male subfertility. Indeed, each bull is usually mated with hundreds of cows maintained in different herds, and the outcome of each insemination is recorded. Bull fertility can therefore be measured accurately in the field and corrected for many confounding factors. Moreover, bull fertility is an important economic trait. Unsuccessful AI can indeed give rise to direct costs, extended calving intervals and increased culling rates of the inseminated cows. Considerable efforts have therefore been made in an attempt to predict bull fertility and to understand the causes of subfertility based on genotypes, semen functional parameters or sperm molecular features, including epigenetic modifications [33, 34]. Some studies on alterations to the sperm methylome in bulls belonging to different fertility classes have recently been published [35–40]. Although they highlighted genes potentially important to fertility and were informative from a biological point of view, all these studies involved small numbers of individuals. These studies also investigated a variety of breeds, considered different phenotypes for fertility, and applied a range of technologies to obtain genome-wide DNA methylation profiles, resulting in marked variations in terms of the magnitude of the DNA methylation changes and the nature of impacted genes. As a result, the link between sperm DNA methylation and male fertility remains poorly understood in cattle, despite all the benefits of the bull model.

In order to contribute knowledge in this field, we investigated the nucleotide-level resolution, genome-wide DNA methylation profiles of semen samples collected from a total of 120 carefully selected Montbéliarde bulls with contrasting fertility levels. We report here on the biological characterization of the loci that were differentially methylated between fertile and subfertile bulls, and on the potential for using these loci to build models predictive of fertility status.

## Results

### Experimental design and overall strategy

**Fig. 1:**
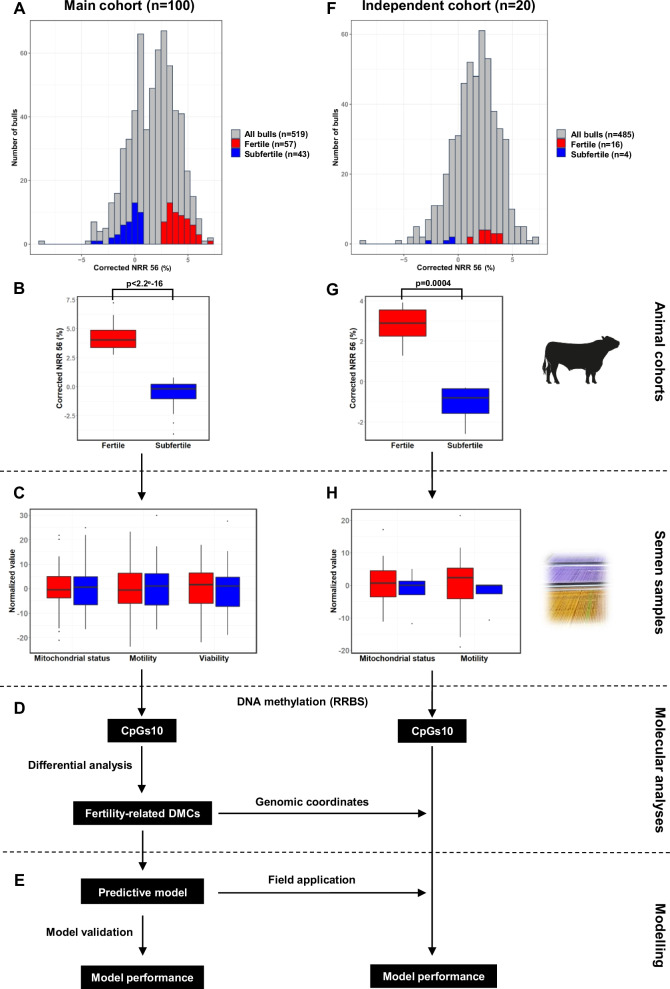
Experimental design and overall strategy. Fertile and subfertile bulls are shown in red and blue, respectively. **A** distribution of the corrected non-return rate at 56 days post-insemination (NRR 56) for all the Montbéliarde bulls born between 2011 and 2014 (519 bulls in total, in grey) and for fertile (*n* = 57) and subfertile bulls (*n* = 43) included in the main cohort. **B** The main cohort comprised 100 bulls with contrasting fertility, based on the differences in corrected NRR 56 (Wilcoxon test, *p* < 0.05). **C** for each bull, straws representing several ejaculates prepared for AI were thawed and pooled. Semen functional parameters corrected for batch preparation effects (see “Methods”) were assessed on these pools and no significant difference in mitochondrial status, motility or viability could be detected between fertile and subfertile bulls (Wilcoxon test, *p* ≥ 0.05). **D** DNA methylation was analyzed on these samples by RRBS, enabling the selection of a subset of CpGs at which DNA methylation could be measured with sufficient precision (CpGs10). These CpGs10 were then subjected to differential analysis and fertility-related DMCs were identified. **E** Using the methylation values at DMCs, a predictive model for fertility was constructed and validated. **F** Distribution of the corrected NRR 56 for all the Montbéliarde bulls born between 2009 and 2012 (485 bulls in total, in grey) and for fertile (*n* = 16) and subfertile bulls (*n* = 4) included in the independent cohort. **G** The independent cohort included 20 bulls with contrasting fertility based on the differences in corrected NRR 56 (Wilcoxon test, *p* < 0.05), and was only used to evaluate the potential for field application of the predictive model built on the main cohort. **H** Semen functional analysis and RRBS were performed on one ejaculate per bull. The model previously built on the main cohort was applied to the methylation values obtained in the independent cohort at CpGs10 identified as DMCs using the main cohort. Model quality indicators assessing the consistency between the actual and predicted fertility were then calculated for both the main and independent cohorts

In order to clarify the relationships between sperm DNA methylation and male fertility and to use variations in the methylome to establish a predictive model, 100 semen samples were obtained from marketed Montbéliarde bulls categorized as fertile (*n* = 57) or subfertile (*n* = 43) according to hundreds of AI records (main cohort, Fig. 1A, B). The bulls were selected based on non-return rates at 56 days (NRR 56), i.e. the proportion of cows that were not re-bred within 56 days of an insemination and could therefore be considered as pregnant. Because these NRR 56 scores were corrected from confounding factors, they fully characterize the bulls and are hardly affected by other sources of variation. The distribution of corrected NRR 56 among all marketed Montbéliarde bulls was normal and quite narrow, and considering its large size and inclusion criteria, the main cohort reflected the most contrasting differences in fertility that could be investigated within this distribution (Fig. 1A). Although the differences in corrected NRR 56 were relatively limited between fertile and subfertile bulls, they were highly significant (Fig. 1B), demonstrating that fertile and subfertile bulls represented two distinct fertility classes.

Several ejaculates representative of the overall fertility of each bull were pooled in order to minimize environmentally or physiologically driven variations that might affect individual ejaculates. To prevent the confounding effect of age on the sperm methylome [41, 42], all ejaculates were collected from animals between 17 and 19 months of age (Additional file 2: Table S1). An assessment of semen functional parameters revealed no statistically significant differences between the fertile and subfertile samples (Additional file 3: Table S2 and Fig. 1C), suggesting that subfertility is not related to defective spermatogenesis. The genome-wide DNA methylation profile of these 100 semen samples was investigated using reduced representation bisulfite sequencing (RRBS) which enabled the identification of fertility-related differentially methylated CpGs (DMCs), which were then annotated and used for functional enrichment analyses and technical validation (Fig. 1D). A Random Forest approach was then applied to the DNA methylation percentages at these DMCs, the aim being to create and validate a model that could predict the fertility class of each bull (Fig. 1E).

The main cohort used to identify DMCs and to build the predictive model was also used to evaluate the model (see “Methods”), which might have led to an overestimation of the model’s performance. To overcome this source of bias and assess the potential of the model for field application, an independent and less controlled cohort that did not contribute to identifying the DMCs was also investigated. This independent cohort comprised 20 individual ejaculates collected from 20 marketed Montbéliarde bulls of contrasting fertility (16 fertile and 4 subfertile) and various ages that had not been included in the main cohort (Fig. 1F, G). Like the main cohort, semen functional parameters did not differ significantly between the fertile and subfertile samples (Fig. 1H; Additional file 3: Table S2). After RRBS analysis, the DNA methylation values at the genomic positions previously identified in the main cohort as DMCs were extracted (Fig. 1D). The predictive model built on the main cohort was then applied to these values in order to classify the samples as fertile or subfertile. Model quality indicators were calculated for both cohorts, based on the consistency between predicted and actual fertility classes (Fig. 1E).

### Reduced representation bisulfite sequencing of 120 semen samples

The sequencing parameters, alignment statistics and overall DNA methylation values for both cohorts were analyzed and compared between fertile and subfertile bulls (Table 1; the asterisks indicate significant differences between fertile and subfertile bulls). Sequencing generated an average of 33.6 million read pairs with an average quality (Phred) score of 38.4, meaning that more than 91% of the sequenced bases had high quality scores. The average bisulfite conversion rate reached 99%. The reads were then aligned on the bovine reference genome. Unique mapping efficiency (34.3% on average) was low but consistent with previous RRBS studies in the bovine species [40, 43, 44]. On average, these uniquely mapped reads aligned on 3.2 million CpGs (out of 28 million CpGs in the genome), of which 60.7% on average were covered by at least 10 reads (CpGs10) and were retained for further analysis. This low unique mapping efficiency was due to the high percentage of reads that aligned at multiple locations on the genome (multimapped reads); this feature was attributed to the large number of repetitive elements targeted by RRBS in the bovine species [43]. Because of their repetitive nature in the genome context, repeats are supposed essentially to be covered by multimapped reads that are filtered-out during bioinformatics analysis. This process results in a loss of information regarding these sequences, which are of functional importance and subject to changes that affect DNA methylation in the sperm of infertile men [27, 45]. In order to obtain additional information on the methylation status of repeats in fertile and subfertile bulls, the reads were also aligned on an artificial genome containing one specimen of each repeat, as defined in Repbase [46]. Although the reads aligned with only 1106 CpGs on average, unique mapping efficiency on this Repbase genome reached an average of 20.7%, translating the stacking of a huge amount of reads at each CpG (an average of 38,341 reads per CpG). There were no statistically significant differences regarding sequencing parameters and alignment statistics on the two genomes when comparing fertile and subfertile bulls from either cohort (Table 1). This eliminated the possibility of technical bias during RRBS library preparation or sequencing that might have affected subsequent results.

**Table 1:** Characterization, mapping efficiency on the bovine reference genome (ARS-UCD2.1) and on a Repbase artificial genome, coverage and average methylation in RRBS libraries

	Main cohort		Independent cohort	
	Fertile (*n* = 57)	Subfertile (*n* = 43)	Fertile (*n* = 16)	Subfertile (*n* = 4)
*Sequencing parameters*				
Number of read pairs (million)	32.9 ± 5.6	33.9 ± 5.4	35.5 ± 5.6	33.9 ± 2.5
Average quality score (Phred score)	38.2 ± 0.3	38.4 ± 0.3	39.0 ± 0.3	38.9 ± 0.4
Bisulfite conversion rate (%)	99.0 ± 0.4	99.0 ± 0.4	99.3 ± 0.6	99.1 ± 0.6
*Alignment on ARS-UCD2.1*				
Uniquely mapped reads (%)	33.9 ± 1.5	34.3 ± 1.6	35.8 ± 2	35.0 ± 3.1
Number of covered CpGs (million)	3.2 ± 0.1	3.2 ± 0.09	3.3 ± 0.1	3.3 ± 0.1
Average coverage per CpG	23.2 ± 3.6	24.0 ± 3.5	24.1 ± 3	22.9 ± 1.8
Percentage of CpGs10	60.2 ± 3.9	60.9 ± 3.5	62.2 ± 2.9	60.3 ± 1.9
Average DNA methylation at CpGs10 (%)	46.2 ± 1.6	46.5 ± 1.2	47.5 ± 1.7	47.8 ± 1.8
Percentage of hypomethylated CpGs10	50.9 ± 2	50.4 ± 1.2	49.3 ± 2	49.0 ± 2.1
Percentage of intermediate CpGs10	6.0 ± 1.1	6.0 ± 0.4	5.8 ± 0.6	6.1 ± 0.2
Percentage of hypermethylated CpGs10	43.4 ± 2.4	43.7 ± 1.1	44.9 ± 1.7	44.9 ± 1.9
*Alignment on RepBase*				
Uniquely mapped reads (%)	20.6 ± 1.7	20.4 ± 1.2	21.6 ± 1.9	22.4 ± 1.6
Number of covered CpGs (million)	1104 ± 72	1124 ± 85	1080 ± 38	1048 ± 4
Average coverage per CpG	36,996 ± 6451	37,722 ± 6606	43,479 ± 8507	43,603 ± 6136
Percentage of CpGs10	70.8 ± 3	70.8 ± 3.1	73.5 ± 2	73.3 ± 2
Average DNA methylation at CpGs10 (%)	30.8 ± 3	31.1 ± 3.2	31.3 ± 2.1	30.6 ± 2.9
Percentage of hypomethylated CpGs10	50.4 ± 2.7	49.5 ± 2.9	49.7 ± 2.9	48.4 ± 5.4
Percentage of intermediate CpGs10	41.3 ± 2	42.1 ± 1.8	40.8 ± 2.5	43.1 ± 5.4
Percentage of hypermethylated CpGs10	8.2 ± 1.9	8.5 ± 2.4	9.4 ± 1.7*	8.5 ± 1.5*

Values are mean ± standard error of the means. CpGs10: CpGs covered by at least 10 uniquely mapped reads. Hypermethylated, intermediate and hypomethylated CpGs10 indicate CpGs10 with average methylation percentages > 80%, [20%; 80%], and < 20%, respectively. Fertile and subfertile bulls were compared in both cohorts, and the asterisks indicate a significant difference in the independent cohort regarding the percentage of hypermethylated CpGs10 (Wilcoxon test, *p* < 0.05)

The average methylation at CpGs10 was 46.5%, which is consistent with values previously obtained on bovine sperm using RRBS [43] and also with the overall undermethylation reported for bovine sperm when compared to adult somatic cells using whole genome bisulfite sequencing [47]. Accordingly, a large proportion of the CpGs10 were hypomethylated (50.4% of CpGs10 with DNA methylation below 20% for the reference genome and 49.9% for the Repbase genome). Except for the percentage of hypermethylated CpGs10 in the Repbase genome, which was slightly reduced in the four subfertile samples from the independent cohort, there were no statistically significant differences between fertile and subfertile bulls in terms of the percentage of CpGs10 that were hypomethylated (DNA methylation below 20%), intermediate (DNA methylation between 20 and 80%) and hypermethylated (DNA methylation higher than 80%), thus indicating that subfertility is not related to global DNA methylation changes (Table 1).

In line with this finding, hierarchical clustering run on the DNA methylation values at CpGs10 failed to segregate samples according to bull fertility (Additional file 1: Fig. S1A). The main cohort included bulls from two semen collection centers and was split into several batches for semen processing and library preparation because of its huge size; we therefore also confirmed that neither the bulls’ origin nor technical artefacts affected the DNA methylation patterns (Additional file 1: Fig. S1B–D).

Taken together, these results show that high quality RRBS data could be obtained regarding DNA methylation analysis in sperm, enabling the investigation of differences related to fertility in the largest cohort so far constituted in cattle.

### Identification of fertility-related differentially methylated CpGs and regions

In order to determine any differences in DNA methylation between the fertility groups in the main cohort, 1,949,735 CpGs10 covered in at least 22 samples per group (which represents half of the smallest group) were subjected to differential analyses using two types of algorithms (see “Methods”). While no DMCs were identified using DSS, which is described as dealing with intra-group inter-individual variability [48], 3252 DMCs were obtained using methylKit [49]. Because sequence polymorphism is an important source of inter-individual variability in DNA methylation patterns [50], this striking difference in the number of DMCs obtained using either DSS or methylKit called for a careful examination of the genomic sequences imputed in the 100 bulls. The presence of polymorphisms affecting C and/or G in the CpG was indeed observed in 2352 out of 3252 methylKit DMCs, suggesting that in our dataset, this algorithm tended to select genetic rather than epigenetic variations. At these sites, the methylation differences between fertility groups were probably emphasized by a biased distribution of genotypes, resulting in a marked intra-group inter-individual variability that precluded their identification as DMCs by DSS but not by methylKit.

To suppress the polymorphisms affecting CpGs that could interfere with DMC detection, all putative variants recorded in the 1000 Bull Genome database and targeting the CpGs covered by RRBS were filtered out, resulting in a background of 1,548,563 CpGs10 covered in at least 22 samples per fertility group (22 samples representing half of the smallest group). Using methylKit, 490 DMCs and 46 differentially methylated regions (DMRs) were identified, which contrasts with no DMCs being obtained with DSS, and suggests that sources of intra-group inter-individual variations still existed in our dataset. This was also visible from the hierarchical clustering run on the CpGs10 before and after the filtering of sequence variants, which demonstrates that inter-individual epigenetic variations unrelated to fertility shape the sperm methylome independently of the presence of polymorphisms (Additional file 1: Fig. S1A, E). These inter-individual epigenetic variations were clearly not due to the presence of variable amounts of somatic cells in the samples (Additional file 1: Fig. S2).

**Fig. 2:**
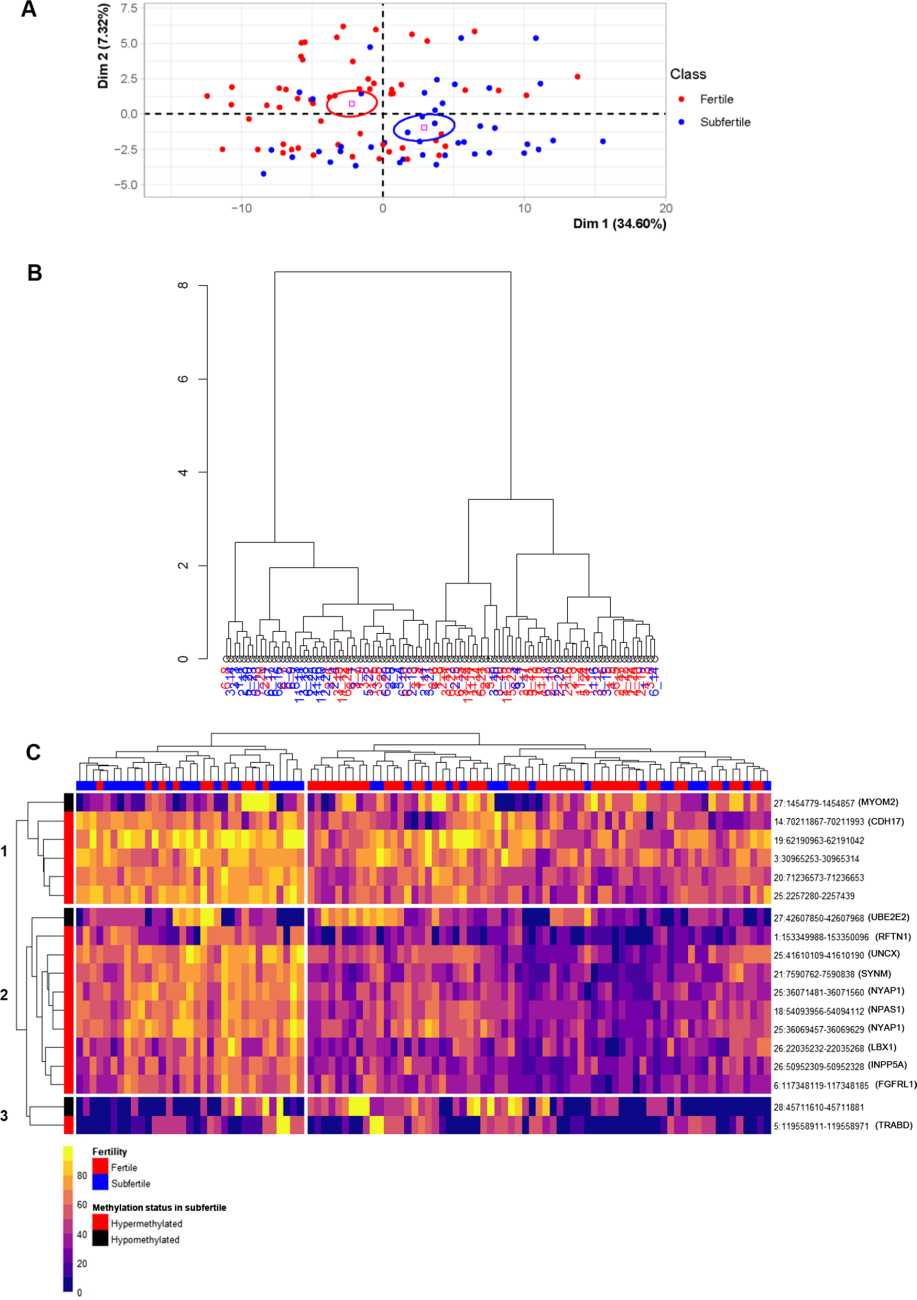
Discrimination of fertile and subfertile bulls based on fertility-related differentially methylated CpGs and regions. **A**, **B** principal component analysis (**A**) and correlation clustering (**B**) run on the DMCs identified after variant filtering between fertile (red) and subfertile (blue) bulls in the main cohort. **A** Although a certain degree of overlap exists between the groups, they clearly segregate along dimension 1. Confidence ellipses are represented. **B** The cluster on the left mostly contains subfertile bulls, while the cluster on the right mostly contains fertile bulls. **C** Heatmap showing the average DNA methylation values at 18 DMRs covered in all 100 samples. Each cell of the heatmap is colored according to the average methylation value in the corresponding sample (displayed in columns, with fertile and subfertile bulls shown in red and blue, respectively) and DMR (in rows, with DMRs hyper- and hypomethylated in subfertile bulls shown in red and black, respectively). For each DMR, the genomic coordinates and the gene containing the DMR (if any) are indicated on the right-hand side. Three different clusters of DMRs were obtained manually. Cluster 1 included 5 DMRs that were highly methylated in subfertile bulls, while cluster 2 included 9 DMRs with low methylation in fertile bulls

Although a certain degree of overlap existed between the fertile and subfertile bulls, they segregated as two distinct groups in a principal component analysis (PCA, Fig. 2A) and in hierarchical clustering run on the DMCs found after variant filtering (Fig. 2B). Likewise, among 18 DMRs covered in all 100 samples of the main cohort, the average DNA methylation status enabled the clustering of samples according to fertility on a heatmap (Fig. 2C). To verify that the overlap between fertile and subfertile bulls was not due to remaining polymorphisms directly targeting DMCs, the imputed genomic sequences in the 100 bulls were examined at DMCs. Only 21 over 490 DMCs (and 5 over the 107 DMCs without missing values used in the PCA) showed the presence of a polymorphism affecting the C and/or the G. Although we cannot definitely rule out that indirect genetic mechanisms could influence DNA methylation at DMCs and lead to this overlap, this result demonstrates that the 1000 Genomes filtering strategy efficiently suppressed most of the variations of methylation artificially due to variants affecting the DMCs. All together, these results suggest that despite the presence of marked inter-individual variability unrelated to fertility at the level of CpGs10, epigenetic information relevant to fertility was embedded in the 490 DMCs and 46 DMRs.

### Fertility-related differentially methylated CpGs and regions target distinct genome features according to their methylation status in subfertile bulls

**Fig. 3:**
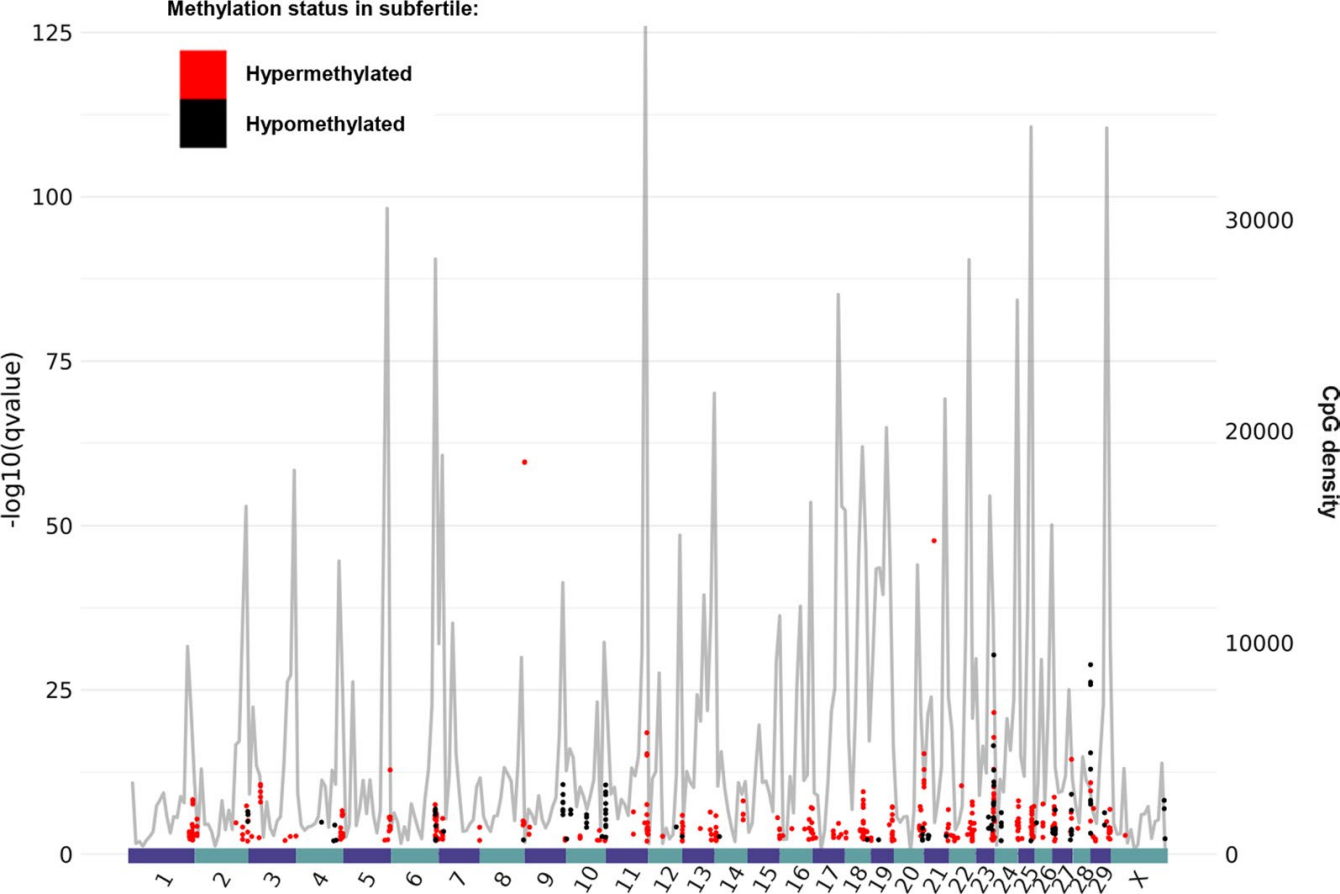
Genomic location and methylation status of fertility-related differentially methylated CpGs. Black and red dots represent DMCs that are hypo- and hypermethylated in subfertile bulls, respectively. The left y-axis indicates a log function of the methylKit *q*-value that reflects the significance of the DMC. Chromosomes are displayed on the *x*-axis. The grey line shows the density of CpGs10 analyzed by RRBS (number of CpGs10 analyzed per window, each window corresponding to the length of the chromosome divided by 300; right *y*-axis). While DMCs hypermethylated in subfertile bulls are scattered throughout the regions targeted by RRBS, hypomethylated DMCs concentrate at discrete and specific regions of the genome

To characterize the 490 DMCs from a genomic point of view, their location was first examined as a function of their methylation status in subfertile bulls versus fertile bulls. Most DMCs were hypermethylated in subfertile bulls (386 out of 490; Additional file 4: Table S3), and these hypermethylated DMCs were found on every chromosome analyzed in the genome (Fig. 3). They were not distributed uniformly but scattered along regions with a dense accumulation of the background CpGs10 (represented by a grey line in Fig. 3), thus indicating no specific enrichment. By contrast, although the DMCs hypomethylated in subfertile bulls only accounted for 21% of all DMCs (104 out of 490), they clustered in discrete regions such as the extremities of chromosome 10 (Fig. 3), which could indicate the presence of restricted domains that tend to lose DNA methylation in subfertile bulls.

Consistent with the fact that most DMCs were hypermethylated in subfertile bulls, 38 out of 46 DMRs were also hypermethylated in subfertile bulls (Additional file 5: Table S4). At the 18 DMRs covered in all 100 samples of the main cohort, most subfertile bulls exhibited a DNA methylation value higher than 50% (orange to yellow, Fig. 2C) while the DNA methylation value of most fertile bulls was close to or below 50% (purple and blue). Of note, among the few DMRs that were hypomethylated in subfertile bulls, two were located on chromosome 10 where stretches of DMCs can be seen in Fig. 3 (Additional file 5: Table S4).

**Fig. 4:**
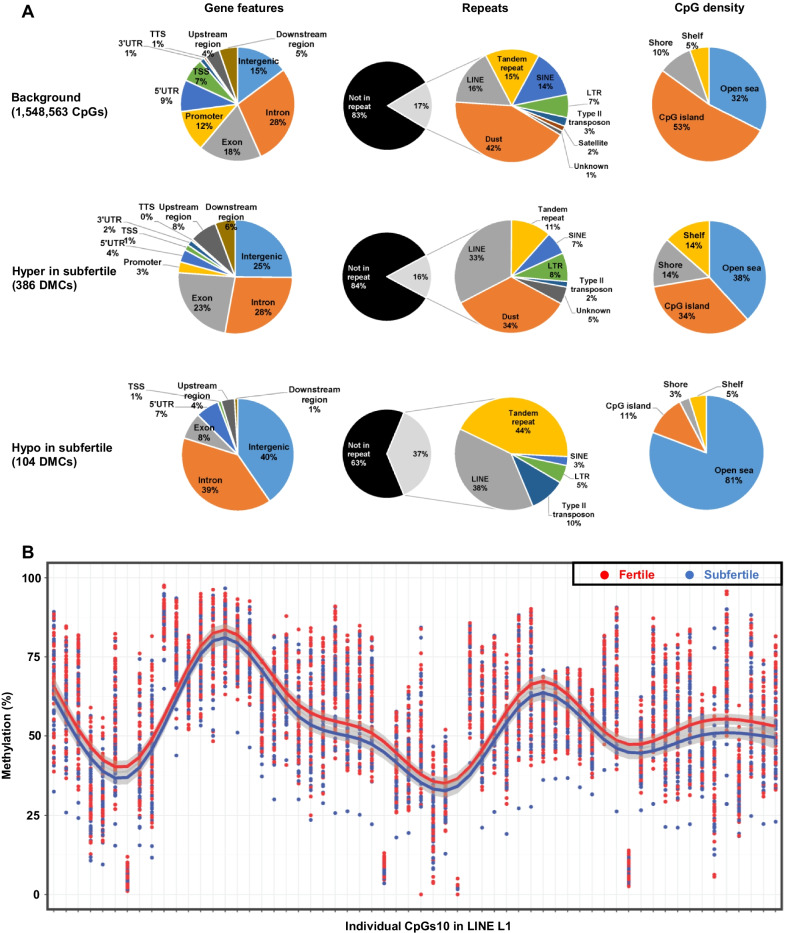
Fertility-related DMCs target different genome features according to their methylation status in subfertile bulls. **A** The background CpGs10 and DMCs were annotated as described in the Methods. The percentage of each genome element in three functional genome features (genes, repetitive elements and CpG islands) is indicated on the different pie charts. DMCs were split into hypo- and hypermethylated DMCs according to their methylation status in subfertile bulls compared to fertile bulls. TSS: transcription start site, TTS: transcription termination site, UTR: untranslated region, upstream region: up to − 10 kb from the TSS, downstream region: up to + 10 kb from the TTS. **B** DNA methylation percentages (y-axis) were obtained for 60 individual CpGs10 included in the consensus sequence of the LINE L1 element (*x*-axis) after the alignment of RRBS sequences on a Repbase artificial genome. Each dot represents one sample, with fertile and subfertile bulls shown in red and blue, respectively. Red and blue lines indicate the trend over the 60 CpGs in fertile and subfertile bulls, respectively, and were obtained using the geom_smooth function of the ggplot2 R library. The 95% confidence interval is shown in light grey. The L1 element was slightly but consistently less methylated in subfertile bulls than in fertile bulls at all CpG positions

The DMCs and DMRs were annotated relative to gene features, repetitive elements and CpG islands, shores and shelves (Additional files 4 and 5: Tables S3 and S4), and the distribution of these different genome features in hypo- and hypermethylated DMCs was analyzed relative to the background. There were only small differences between the DMCs hypermethylated in subfertile bulls and the background (Fig. 4A, upper and middle panels), such as an enrichment in intergenic sequences, in long interspersed nuclear elements (LINEs) and in shelves; in parallel, there was a depletion of promoters, transcription start sites (TSSs), 5′ untranslated regions (5′UTRs) and CpG islands (CGIs). These results were quite similar to the genome features targeted by the 46 DMRs, most of which were made up of DMCs hypermethylated in subfertile bulls (Additional file 1: Fig. S3). The differences between DMCs hypomethylated in subfertile bulls and the background were more marked (Fig. 4A, upper and lower panels). Indeed, there was a clear enrichment of DMCs in intergenic sequences and depletion in exons, promoters and TSSs, which paralleled an overrepresentation of DMCs present in open sea relative to regions dense in CpGs (CGIs, shores and shelves). A striking enrichment in repetitive elements, and particularly in tandem repeats and LINEs, was also observed among these hypomethylated DMCs.

To further analyze these repeats, a differential analysis was performed between fertile and subfertile bulls using the methylation values obtained after alignment on the Repbase genome (Additional file 6: Table S5). This approach failed to highlight any CpGs10 displaying significant differential methylation with respect to bull fertility. However, individual CpGs10 located in the consensus LINE L1 consistently exhibited lower DNA methylation in subfertile bulls when compared to fertile bulls (Fig. 4B). Furthermore, the average DNA methylation of LINE L1, but not of LINE BovB, tended to be slightly decreased in subfertile bulls (Additional file 1: Fig. S4A), thus supporting the finding that DMCs hypomethylated in subfertile bulls are enriched in LINEs. The principal reason why no CpG in LINE L1 was found to be differentially methylated was due to the methylation difference between groups (2.7% on average), which was lower than the threshold of 10% set for the methylKit analysis. The same approach was applied to other families of repetitive elements referenced in Repbase, but no differences could be found between fertile and subfertile bulls, as exemplified by members of the long terminal repeat (LTR) family (Additional file 1: Fig. S4B).

To conclude this section, DMCs and DMRs hypermethylated in subfertile bulls accounted for most of the methylation differences found between the two fertility groups, and targeted genome features distinct from those targeted by hypomethylated DMCs and DMRs. Interestingly, LINEs were enriched in both hypermethylated (33% vs. 16% LINEs in the background) and hypomethylated (38% vs. 16%) DMCs. However, at least for LINEs in the L1 family, a trend toward hypomethylation in subfertile bulls was observed using a Repbase genome that has the potential to capture information from the multimapped reads.

### Differentially methylated genes are related to development and sperm physiology

**Fig. 5:**
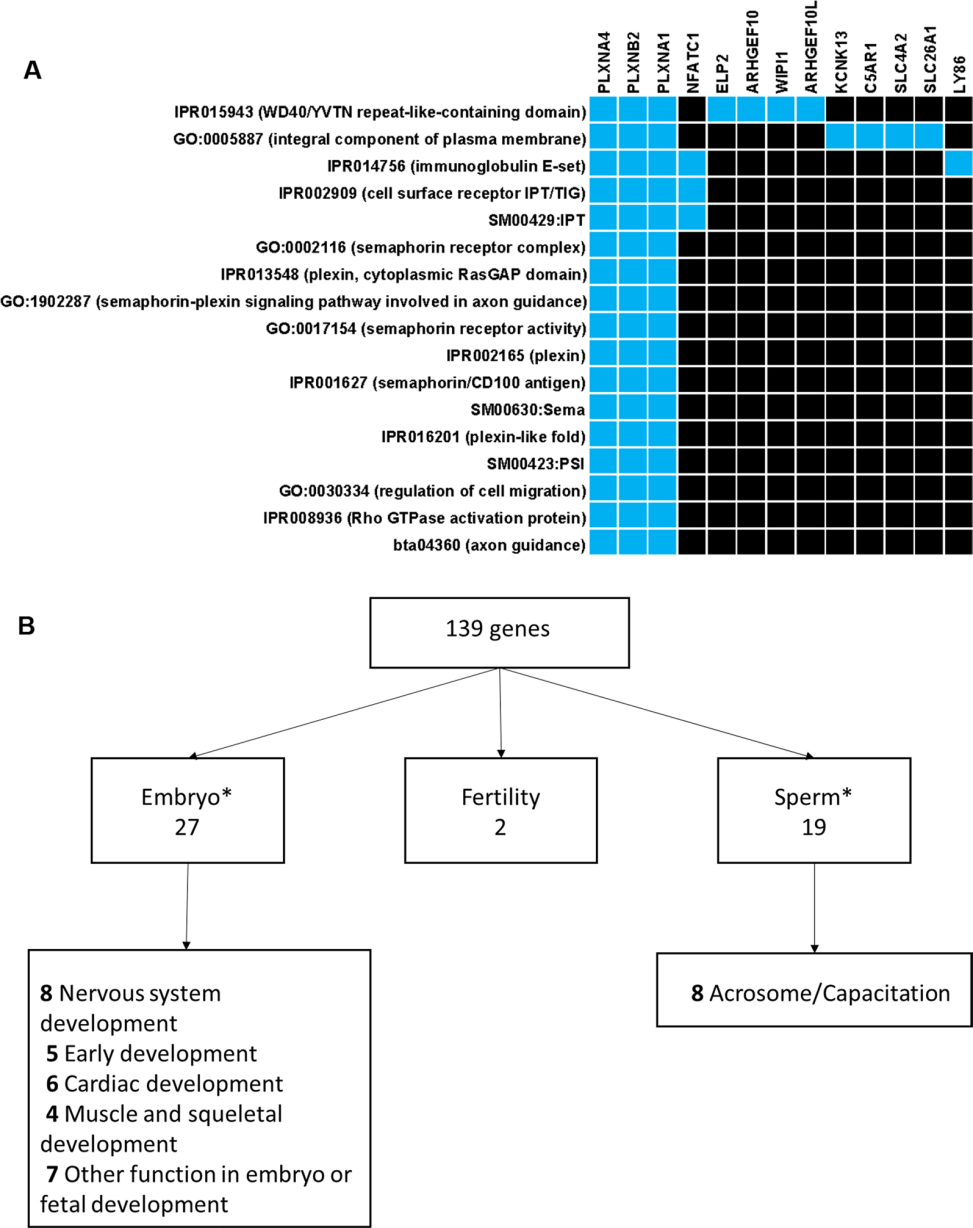
Differentially methylated genes are relevant to male fertility. **A** functional enrichment analysis was performed using DAVID, where genes containing DMCs (170 genes) were compared to those covered in the background CpGs10 (19,829 genes). The enrichment cluster with an EASE score of 1.84 is represented, with genes in the cluster displayed on the x-axis and the different terms on the *y*-axis. A correspondence between a gene and a term is indicated by a light blue color on the heatmap. **B** A systematic review of the literature was undertaken for all genes associated with DMCs that contained a gene name (*n* = 139). The NCBI Pubmed database was interrogated using the gene names successively associated with each of the following terms: “embryo*”, “sperm*” and “fertility”. Some genes matched with several terms and are represented in several categories, explaining why the total number of counts exceeded the number of unique genes of interest (46 unique genes)

The next step was to analyze the biological function of the genes targeted by differential methylation between fertile and subfertile bulls. For this purpose, a functional enrichment analysis and an extensive review of the literature were conducted on the 170 genes containing at least one DMC in genic regions and/or in upstream or downstream regions (up to 10 kb from a gene). To increase the number of genes in the analysis at this step, all DMCs were considered independently of their hypo- or hyper-methylation status in subfertile bulls. A Database for Annotation, Visualization and Integrated Discovery (DAVID) analysis highlighted a unique cluster of enrichment that reached significance (EASE score of 1.84, 1.3 being the lower limit for significance; Fig. 5A). Three genes located on three different chromosomes, *PLXNA4* (chromosome 4), *PLXNB2* (chromosome 5) and *PLXNA1* (chromosome 22)*,* which belong to the Plexin family, were associated with all the terms in the cluster. Plexins are present on the surface of cells and interact with the semaphorin family of proteins to trigger a rearrangement of the cytoskeleton and to regulate cell shape, differentiation, junctions, motility and survival. All these processes are involved in the remodeling of epithelia and endothelia during organogenesis as well as in axon guidance during development of the nervous system [51].

To go further, we performed a systematic review of the literature targeting the differentially methylated genes. Genes relevant to development, sperm function and fertility accounted for a large share of all differentially methylated genes (46 unique genes out of 139; 33%) and are listed, together with the corresponding references, in Additional file 7: Table S6. The largest share of these 46 unique genes was involved in embryonic and fetal development (Fig. 5B). It is noteworthy that genes involved in nervous system development were highly represented, which was consistent with the results of the DAVID analysis. By contrast, only five genes were related to early embryonic development*,* which concern cytokinesis, blastocyst formation and gastrulation. As for sperm function, genes related to the formation of the acrosome and capacitation represented eight out of 19 genes. Finally, two genes were associated with male and female fertility in genome-wide association studies in cattle.

We next compared the 139 genes differentially methylated between fertile and subfertile bulls with genes differentially methylated between cases and controls in four human studies investigating male fertility [26–28, 52]. Twenty-five differentially methylated genes were found in common with at least one human study, among which 18 genes exhibited a consistent methylation status between our study and the human studies (Additional file 8: Table S7). Out of these 25 genes, 17 were not highlighted during the systematic literature mining we performed (Additional file 7: Table S6). Because they were differentially methylated as a function of fertility in two different species, these 17 genes may be additional candidates relevant to male fertility.

In conclusion, differential methylation between fertile and subfertile bulls targeted genes with reported functions in sperm physiology, differentiation and post-testicular maturation, early and post-implantation development and processes important to organogenesis and nervous system development, all being relevant to male fertility.

### Validation by bisulfite pyrosequencing

The quantification of DNA methylation using RRBS is reliant on the counts of reads bearing “C” and “T”, which can lead to a certain degree of imprecision and to sampling biases, especially for CpGs where coverage is limited [53]. The quantification of DNA methylation at some CpGs using an independent technique was therefore necessary to validate both the molecular and bioinformatics aspects of the RRBS results.

**Fig. 6:**
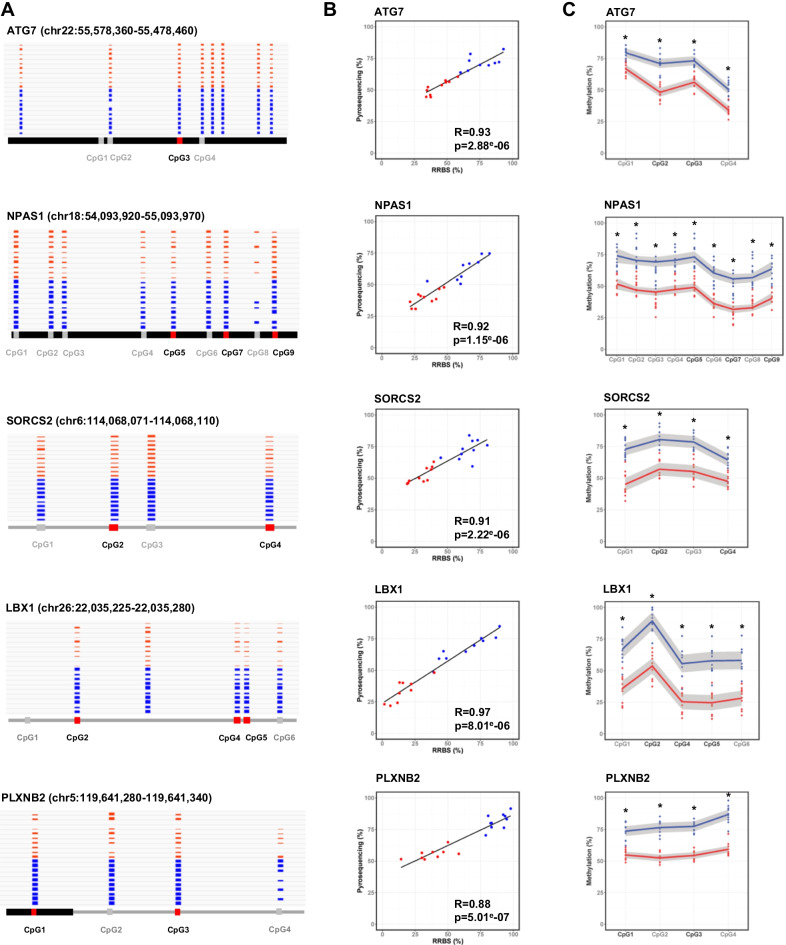
Bisulfite-pyrosequencing validations on twenty fertile and subfertile semen samples. **A** IGV browser views of the regions targeted for pyrosequencing in the *ATG7, NPAS1, SORCS2, LBX1* and *PLXNB2* genes. The red and blue bar charts represent the methylation percentages at each CpG10 position for fertile (*n* = 10) and subfertile (*n* = 10) bulls, respectively. The CpGs analyzed by pyrosequencing are numbered according to their 5′–3′ position along the genome. The CpGs identified as fertility-related DMCs are indicated in black text and red boxes, while non-DMCs are indicated in grey. **B** For each gene region, the average methylation percentage measured by pyrosequencing (*y*-axis) was calculated for the DMCs included in the region and plotted against the average methylation percentage measured by RRBS at the same DMCs (*x*-axis). Each dot represents one sample from the fertile (in red, n = 9 to 10) and subfertile (in blue, *n* = 9–10) groups. The least squares lines of best fit and Spearman’s rank R correlation coefficients are indicated. All correlations were highly significant (Spearman’s rank correlation test; *p* < 0.05). **C** Methylation percentages of individual CpGs assayed by pyrosequencing in fertile (in red, *n* = 10) and subfertile (in blue, *n* = 10) bulls. CpGs are numbered according to **A** and DMCs are highlighted in black. Dots show the methylation levels of individual samples, while the trends per fertility group are indicated by red and blue lines obtained using the geom_smooth function of the ggplot2 R library, together with 95% confidence intervals in light grey. Asterisks indicate that the methylation percentage measured by pyrosequencing differed significantly between fertility groups for all analyzed CpGs (Wilcoxon test, *p* < 0.05)

Here, 11 DMCs located in five genes relevant to development (*PLXNB2, NPAS1, LBX1* and *SORCS2*) and sperm function (*ATG7*) were validated using bisulfite pyrosequencing (Fig. 6). These DMCs are located in diverse genomic contexts, such as gene upstream regions (*LBX1,* Additional file 1: Fig. S5), exons (*NPAS1*), introns (*SORCS2*), intron–exon junctions (*PLXNB2*) and 3′ UTRs (*ATG7*), and reflected the overall hypermethylation of DMCs in subfertile bulls when compared to fertile bulls (Fig. 6A). Because the main cohort was too large to be fully analyzed with the low-throughput system used for pyrosequencing, ten samples with contrasting levels of methylation were selected in each fertility category for each gene (Additional file 9: Table S8). For all genes, the average DNA methylation values at DMCs obtained using RRBS and bisulfite-pyrosequencing were significantly correlated (Spearman’s correlation coefficient between 0.88 and 0.97; *p* < 0.05), thus validating our RRBS data from a technical perspective (Fig. 6B). As expected, pyrosequencing confirmed the significantly higher DNA methylation level in subfertile bulls at all the DMCs investigated (Fig. 6C).

Pyrosequencing enabled the quantification of 15 additional CpGs surrounding DMCs. Interestingly these CpGs displayed the same behavior as nearby DMCs, being significantly more methylated in subfertile bulls in the pyrosequencing data (Fig. 6C). This trend could also be seen in the RRBS data (Fig. 6A) and was in line with the many reports showing that CpGs located within the same region tend to behave in the same way [54–56]. Despite the similar behavior of nearby CpGs, of the five genes we analyzed more closely only *LBX1* and *NPAS1* contained a DMR; more generally, only 225 out of 490 fertility-related DMCs clustered into DMRs. These results may be linked to the maximal inter-DMC distance required to constitute a DMR (see “Methods”), which could be a limitation under an RRBS approach that covers discontinuous portions of the genome [57]. Another reason may have been the insufficient coverage of nearby CpGs which were therefore not integrated into the background and did not undergo differential analysis.

Taken together, the bisulfite-pyrosequencing results (1) were strongly aligned with the RRBS findings; (2) confirmed the hypermethylated status of DMCs in subfertile bulls compared to fertile bulls for five genes relevant to male fertility, and (3) suggest that the number of DMRs identified during our analysis may have been underestimated because of the stringency of the bioinformatics settings. Fertility-related DMCs could therefore be used with confidence in subsequent steps of our study.

### Bull fertility status can be predicted from the sperm methylome using a Random Forest approach

**Fig. 7:**
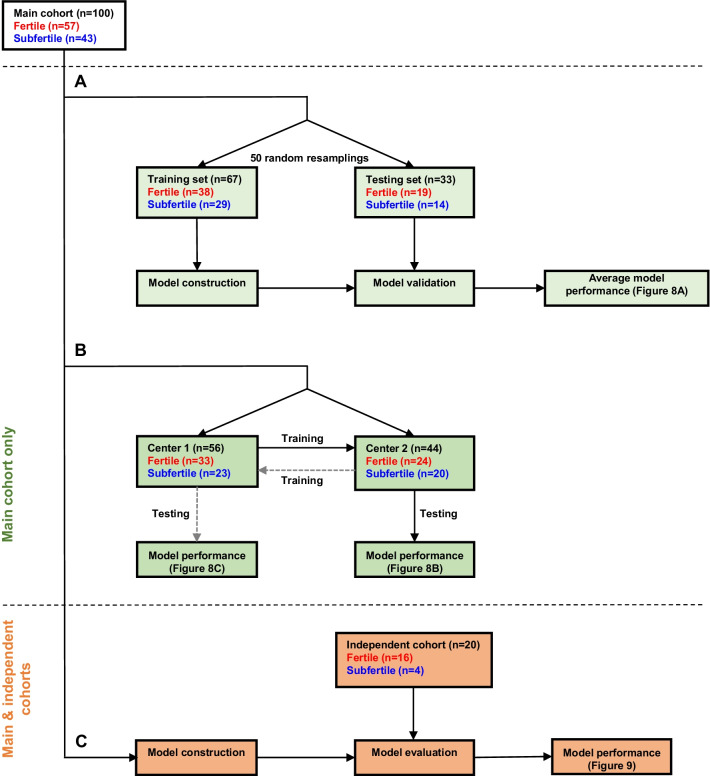
Strategies to evaluate the performance of models predictive of fertility status. **A** model construction and cross-validation. The main cohort was split into a training set and testing set. The training set contained 2/3 of the samples (*n* = 67) randomly selected but with conservation of the original proportion of fertile and subfertile samples, and was used to create the model. The testing set contained 1/3 of the remaining samples (*n* = 33) and was used to evaluate the predictive ability of the model previously constructed on the training set. This process was iterated 50 times with a resampling of the training and testing sets, and the model performance was averaged over these 50 iterations. **B** Effect of the bulls’ origin on the model performance. The main cohort was split into a semen collection center 1 set (*n* = 56) and a semen collection center 2 set (*n* = 44), which were then used alternatively to train and test the predictive model. **C** Potential of the model for field application, using an independent and less controlled cohort. The whole main cohort was used to train the model, which was then tested on the independent cohort

**Fig. 8:**
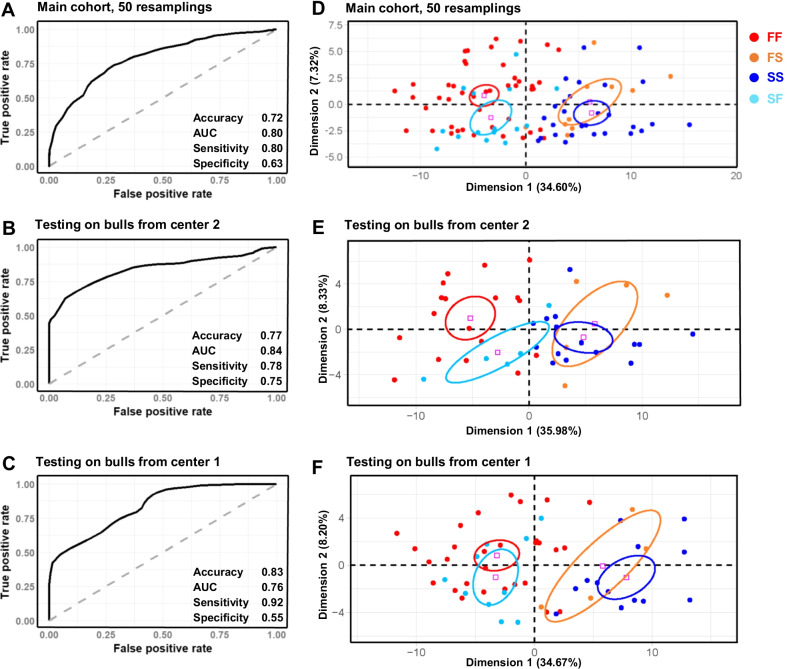
Fertility in the main cohort can be predicted from DMCs. **A**–**C** Random Forest models were built on three different training sets from the DNA methylation values of 107 fertility-related DMCs without missing values. The models were then run on three different testing sets, and predicted fertility status was compared against the actual fertility status of bulls included in each type of testing set. Receiver operating characteristics (ROC) curves and four performance indicators are displayed: accuracy (correct prediction rate), area under the ROC curve (AUC), sensitivity (true positive rate) and specificity (true negative rate). **D**–**F** For each model, principal component analysis was run on the 107 fertility-related DMCs and an individual factor map is displayed, with bulls colored according to the consistency between their predicted and actual fertility status (red: fertile predicted as fertile, FF; blue: subfertile predicted as subfertile, SS; orange: fertile predicted as subfertile, FS; light blue: subfertile predicted as fertile, SF). Confidence ellipses are indicated. **A**, **D** the main cohort was split into a training set of 67 bulls and a testing set of 33 bulls, the proportions of fertile and subfertile bulls being the same in each set. The average performance indicators were calculated after 50 iterations with a random resampling of the training and testing sets. **B**, **E** The training set included bulls originating from semen collection center 1 while the testing set included bulls originating from semen collection center 2. **C**, **F** The training set included bulls from center 2 while the testing set included bulls from center 1. Whatever the model, the performance indicators were within the same range; misclassified bulls were found in both fertility classes and exhibited a DNA methylation pattern typical of the opposite class

Bull fertility was next modelled using the DNA methylation values obtained on the main cohort at 107 DMCs with no missing values (Additional file 4: Table S3). For the model construction and validation, the cohort was split randomly into a training set (2/3 of the animals) and a testing set (1/3) with the same proportion of fertile and subfertile bulls as in the original dataset (Fig. 7A). Using a Random Forest approach, which is described as suitable for molecular biology data [58], a model was then built using the training set, and the prediction was assessed by comparing the estimated fertility and actual fertility of the testing set. The average performances of the model were calculated from 50 iterations of this process, with resampling of the training and testing sets at each iteration. This cross-validation indicated satisfactory performance, with values for the area under the receiver operating characteristics (ROC) curve (AUC) and accuracy of 0.80 and 0.72, respectively (Fig. 8A). The model displayed higher sensitivity (0.80) than specificity (0.63), which means that errors were most frequently caused by a misclassification of subfertile bulls.

Because the sperm methylome is sensitive to a wide range of environmental variations [59] there is a potential risk that DNA methylation at fertility-related DMCs may vary beyond fertility, which could alter the predictive performance of the model that was developed using these DMCs. The main cohort included bulls that were commercialized by two breeding companies, maintained in two different semen collection centers 100 km distant from each other and under different animal management practices. This experimental design offered an opportunity to assess the degree to which the performance of the model was affected by the origins of the bulls (Fig. 7B). Using the 107 DMCs with no missing values, a new Random Forest predictive model was built from the methylation values measured on the 56 samples collected in center 1 and tested on the 44 samples collected in center 2 (Fig. 8B), and vice-versa (Fig. 8C). Satisfactory performance was achieved in both cases, with accuracies of 0.77 and 0.83, and AUCs of 0.84 and 0.76, respectively. Sensitivity and specificity varied more markedly and in opposite directions. Indeed, the model trained on the samples collected in center 1 and tested on samples collected in center 2 was best for the correct prediction of subfertile bulls (specificity of 0.75). By contrast, the model trained on the samples collected in center 2 and tested on samples collected in center 1 failed to correctly predict subfertile bulls (specificity of 0.55) but outperformed in terms of the correct prediction of fertile bulls (sensitivity of 0.92). This result thus demonstrated that the model’s performance was only moderately impacted by the origins of the bulls, suggesting the robustness of the model regarding a certain degree of environmental variation.

In order to understand why 20–30% of the bulls were systematically misclassified in our model, PCA was run on the DNA methylation values measured at DMCs, and individuals were classified as fertile bulls predicted as being fertile (FF), fertile bulls predicted as being subfertile (FS), subfertile bulls predicted as being subfertile (SS) and subfertile bulls predicted as being fertile (SF). Very similar results were observed with the three models: fertile and subfertile bulls that were correctly predicted were discriminated by the first dimension of PCA, while misclassified bulls tended to cluster with the opposite fertility class (Fig. 8D–F). This result clearly showed that at DMCs, the 20–30% misclassified bulls displayed a DNA methylation signature typical of the opposite class, thus producing the wrong predictions. Several factors were therefore investigated in order to relate these unexpected DNA methylation patterns to biological or technical features specifically affecting the misclassified bulls. No significant differences between misclassified and correctly classified bulls in both fertility classes were observed regarding fertility (Additional file 1: Fig. S6A), which indicated that although they displayed a DNA methylation pattern that mimicked that of fertile bulls, the misclassified subfertile bulls were indeed subfertile; the reverse was also found for misclassified fertile bulls. Moreover, a later fertility indicator (sire conception rate, SCR) further confirmed their correct assignment to the two fertility classes (Additional file 1: Fig. S6B), which was initially performed according to the NRR 56 (Additional file 1: Fig. S6A). No significant differences between the FF, FS, SF and SS groups were found regarding the number of AIs used to measure fertility (Additional file 1: Fig. S6C) and semen functional parameters (Additional file 1: Fig. S6D–F). The four groups were also equivalently distributed in terms of the season of semen collection, number of ejaculates per sample and technical batches (Additional file 1: Fig. S6G–J), thus demonstrating the absence of confounding factors in our experimental design. In the absence of any identified source of bias, it is therefore possible that the fertility of misclassified bulls was independent from DNA methylation at these 107 DMCs. To extend the panel of CpGs, we tried to impute missing values and built a model using 295 DMCs, but this did not cause any significant change to the results (Additional file 1: Supplementary Results and Additional file 1: Fig. S7).

**Fig. 9:**
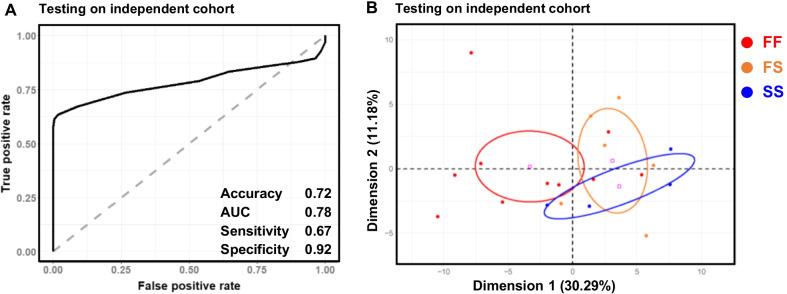
Fertility in an independent cohort can be predicted from DMCs. **A** a Random Forest model was built on the main cohort using the DNA methylation values of 107 fertility-related DMCs without missing values. The model was then run on the independent cohort, and the predicted fertility status was compared with the actual fertility status. ROC curves, accuracy, AUC, sensitivity and specificity were comparable to the performance indicators obtained when the testing set was included in the main cohort. **B** principal component analysis was run on the 107 fertility-related DMCs, and an individual factor map is displayed, with bulls colored according to the consistency between their predicted and actual fertility status (red: fertile predicted as fertile, FF; blue: subfertile predicted as subfertile, SS; orange: fertile predicted as subfertile, FS). Confidence ellipses are indicated. Misclassified bulls were found in the fertile class only and displayed a DNA methylation pattern similar to that of the subfertile class

The performance of the model was next assessed on bulls in the independent cohort that had not been used to identify DMCs (Fig. 7C). It should be noted that this independent cohort was less controlled in terms of the age of the bulls, which ranged from 15 to 39 months, and each bull was only represented by one ejaculate, since the pooling of several ejaculates would clearly limit the practical applications of our study. The accuracy and AUC of the model on this independent cohort (respectively 0.72 and 0.78 in Fig. 9A) were comparable to those found using the main cohort, suggesting that practical applications of the model could be envisioned in field conditions. Unlike the results described above, sensitivity was, however, lower than specificity, which indicates that the misclassifications resulted from fertile bulls predicted as being subfertile. A PCA was run on the methylation values of the independent cohort and, as previously observed with the main cohort, FS bulls clustered separately from FF bulls (Fig. 9B), suggesting again that the fertility of misclassified bulls was independent of DNA methylation. Unlike the main cohort, no misclassification was observed for subfertile bulls. This result may have been related to the unbalanced number of bulls in each class (*n* = 4 subfertile bulls vs. *n* = 16 fertile bulls), which was closer to the incidence of subfertility in the whole population than that seen in the main cohort.

Taken together, these results demonstrate that for approximately 75% of the bulls, field fertility could consistently be predicted from the sperm DNA methylation status of 107 CpGs, whatever the origin and age of the bulls and the number of ejaculates in the sample. For 25% of the bulls belonging to both fertility classes, prediction was hampered by a reversed DNA methylation status at these CpGs, but also at an extended panel of 295 CpGs. In the absence of any identified source of bias, these results suggest that the fertility of a subset of bulls was independent of DNA methylation, at least at the CpGs investigated.

## Discussion

This study was performed on the largest cohort so far used to investigate the relationships between male fertility and DNA methylation in cattle, and one of the largest cohorts of any mammals, including humans. Field fertility was very precisely assessed for 120 bulls using two different indicators and we also demonstrated that the experimental design was devoid of any obvious confounding effects. Using this valuable resource, we generated high quality and technically validated genome-wide DNA methylation data using RRBS. Our main finding was that although they did not differ in terms of semen functional parameters at 17–19 months of age, fertile and subfertile bulls displayed subtle DNA methylation differences in the same semen samples, and these differences could be used successfully to build models predictive of fertility status for the whole career of the bulls.

### Inter-individual variability of the sperm DNA methylome

One striking finding revealed by our analysis was the important inter-individual variability observed in the sperm methylome at both the genome-wide and DMC levels. This inter-individual variability, which could be appreciated for the first time in cattle thanks to the large size of the main cohort, was independent of fertility and impacted all the results of our study. It firstly precluded the identification of DMCs using a stringent algorithm such as DSS [48], leading to a certain degree of overlap between fertile and subfertile bulls at the DMCs identified using the less stringent algorithm methylKit [49]. Heterogeneity at DMCs then slightly altered the performance of the predictive model, as it led to the misclassification of 20–30% bulls displaying a DNA methylation profile typical of the opposite class.

Inter-individual variability was not related to variations affecting individual ejaculates, since each bull was represented by several ejaculates. Because the automation of DNA methylation assays has been reported to improve reproducibility [60], we used a partially automated process to generate libraries, and checked that technical artefacts did not confound our results at each step of data generation. The sequencing parameters thus varied within the same range for the two fertility groups, and the RRBS results were not affected by semen processing and library preparation batches. Moreover, the excellent correlations between the RRBS and bisulfite-pyrosequencing results demonstrated that the technical limitations inherent to RRBS did not account for inter-individual variability in our data, and confirmed that our study was adequately powered in terms of sample size and sequencing depth to detect small differences between groups [53]. Genetic factors are another important source of variation in DNA methylation patterns [50], and it has been proposed that both genetic factors and somatic cell contamination confound DNA methylation analyses in human sperm [31]. Although we cannot definitively rule out the possibility that genetics interfered with our results via indirect mechanisms, we limited this effect by filtering out putative variants from the CpGs we analyzed. We also checked that the DNA methylation results were not altered because of residual contamination by somatic cells.

Because the effects of most technical factors could be regarded as insignificant in our study, one possibility is that stochastic, indirect genetic effects or uncontrolled environmental or physiological factors underlie inter-individual variability in the sperm methylome [61]. Among the physiological factors that have been described to impact the sperm methylome, age has a major effect in cattle [41, 42], humans [62] and mice [63], but could be excluded here as only ejaculates collected from bulls aged between 17 and 19 months were used. Because the sperm epigenome is also responsive to a wide range of environmental factors [59], we confirmed that the bulls’ origins with respect to semen collection center did not interfere with the RRBS results and model performance. However, bulls are usually maintained in various herds before being recruited by a breeding company, and these diverse conditions may have modified the sperm methylome as a function of each individual bull’s environment, finally affecting fertility without inducing a homogenous DNA methylation signature. Male germ cells are indeed subject to intense epigenetic remodeling during in utero and early post-natal life, and the vulnerability of the methylome to environmental variations during these periods may alter fertility [11, 12, 64]. Another common practice that might also affect the sperm methylome is that once arrived in a station, male calves are fed in order to optimize their average daily gain. The sperm epigenome is sensitive to nutrition [9] and modifications to early life nutrition in cattle have been reported to induce persistent changes to the sperm methylome [65]. The metabolic response of each bull to this practice may therefore vary as a function of its genetics and initial body condition, leading to epigenetic inter-individual variability. Finally, because it has also been suggested that heat stress might alter bull fertility through epigenetic mechanisms [66], we investigated the effects of the season of ejaculate collection on our experimental design. The distribution of fertile and subfertile bulls in both hot and cold seasons, whatever their methylation pattern and the outcome of the prediction, means that a risk of heat stress confounding the overall results of our study is unlikely, but does not completely exclude that it contributed to a certain degree of inter-individual variability. Therefore, although we controlled numerous factors likely to interfere with fertility-related variations in the sperm methylome, uncontrolled factors still remained in our study. Because humans live under far less standardized conditions and display more genetic diversity than cattle, it is probable that the magnitude of inter-individual variability is even more important in the human sperm methylome. Of all the confounding factors present in humans, the effect of this epigenetic inter-individual variability on the consistency of the results reported on male fertility may have so far been overlooked [29, 32].

### Prognostic value of the sperm DNA methylome for male fertility

Despite the important inter-individual variability we observed in our methylation data, the majority of the bulls displayed a DNA methylation profile at DMCs that enabled the creation of a predictive model with satisfactory performance. Importantly, this model displayed comparable performance regardless of the bulls’ origin, and also when tested on individual ejaculates from an independent cohort that included bulls of various ages, thus demonstrating its robustness to certain variations in environmental or physiological factors. Interestingly, fertility status could be successfully predicted for all individuals with a DNA methylation pattern typical of their fertility class at DMCs, which demonstrated the potential of Random Forest approaches to model phenotypic outcomes from DNA methylation.

To our knowledge, this study represents the most comprehensive attempt to model bull fertility from the sperm methylome, which was probably enabled by the large size of the main cohort. Inadequate sample size has indeed been proposed as a major obstacle to replicating fertility-related DNA methylation signatures in human sperm [29]. In line with this view, the important inter-individual variability we report here makes it unlikely that the methylation differences previously reported between small numbers of bulls of contrasting fertility (*n* = 3–9 per group; [35–40]) can reflect those in a larger population with less extreme differences in fertility, thus limiting modelling approaches adapted from these datasets. Only Takeda et al. [39] confirmed the differential methylation between low and high fertility bulls using a wider population (*n* = 50) at 10 loci and were able to construct a predictive model that has now to be assessed on an independent cohort.

In our model, 20–30% of bulls with divergent DNA methylation profiles were consistently misclassified whatever the testing set used, underscoring the importance of a thorough assessment of models using independent populations. Of note, comparable model performance was also achieved using an extended panel of DMCs after the imputation of missing values. From a statistical point of view, this observation suggests that whatever the number of variables in the model, DNA methylation at DMCs does not suffice to explain the whole variance related to fertility. It is possible that the relatively limited differences in fertility that exist between fertile and subfertile bulls, together with the important inter-individual variability we report, may have precluded the identification of more discriminant DMCs from which the percentage of misclassifications would have been weaker. Importantly, both fertile and subfertile bulls were found to be misclassified, which indicates that a DNA methylation profile typical of fertile bulls is not sufficient to be fertile, which might be expected given the multifactorial nature of fertility. More surprisingly however, a DNA methylation profile typical of subfertile bulls does not necessarily lead to subfertility, suggesting the existence of compensatory mechanisms. In line with this finding, a study investigating the association between the sperm methylome and fecundity in humans reported that only 54% of men displaying the unfavorable DNA methylation pattern actually failed to conceive [28]. To improve predictive performance, a future direction might be to build the model using a less biased selection of CpGs than that resulting from a between-group differential analysis, as DNA methylation patterns at fertility-related DMCs are not conserved in all individuals. A further step would be to integrate other types of epigenetic features that might capture complementary aspects of the variance related to male fertility.

### Biological features targeted by differential methylation

From a biological perspective, the comparable model performances obtained on the main and independent cohorts demonstrated that at DMCs, fertility-related variations of DNA methylation could be replicated in a significant proportion of animals that were completely independent from DMC identification. The biological features associated with these DMCs therefore represent a valuable source of information that could help to improve our understanding of male fertility.

Among the 139 genes targeted by DMCs, 19 were found to be important for sperm physiology, differentiation and post-testicular maturation, which is obviously of considerable interest regarding fertility. For instance, *ATG7*, on which we focused during the bisulfite-sequencing validation, is involved in autophagy and associated with formation of the acrosome and with spermiogenesis [67]; impairment of these functions in *ATG7* −/− germ cell-specific mice drive complete infertility [68]. Although several genes related to acrosome function were found to be differentially methylated in our study, it is unlikely that the subfertile bulls suffered from major spermiogenesis defects, which would probably have led to a more severe phenotype than that observed. However, in light of the phenotype described in *ATG7* −/− germ cell-specific mice, it would be interesting to analyze the acrosome function in these bulls in greater detail.

During our study, 27 genes involved in development, 8 of them related to nervous system development, were also found to be differentially methylated between fertile and subfertile bulls. This finding agreed well with the absence of major alterations to semen functional parameters in subfertile bulls, as impaired development could lead to loss of the embryo and subsequently to a reduction in fertility without necessarily affecting semen functional parameters. We focused in particular on three genes, which were further analyzed by bisulfite-pyrosequencing: *NPAS1*, *LBX1* and *SORCS2*. *NPAS1* is a member of the basic helix-loop-helix per-ARNT-SIM (bHLH-PAS) family of transcription factors. Several genes in this family are known to be involved in nervous system development [69]; *NPAS1* in particular has been related to fertility in a genome-wide association study conducted on cattle [70]. *SORCS2* encodes a VPS10 domain-containing receptor that is highly expressed in developing neural tissues [71, 72], and *LBX1* is a homeobox gene orthologous to the *ladybird* gene in Drosophila, which is important for limb, neural and heart development [73–76]. A DAVID analysis also highlighted three genes involved in axon guidance*: PLXNA1, PLXNA4, PLXNB2* (which we further analyzed by bisulfite-pyrosequencing). Strikingly, these three genes encode plexins of different classes that are located on different genome regions*,* suggesting the functional importance of this protein family to bull fertility. Plexins are receptors of semaphorins and both types of protein belong to the semaphorin signaling pathway that regulates cell adhesion, migration, division, differentiation and survival, acting on a wide range of developmental processes [51]. *PLXNB2* in particular is highly expressed in neuronal progenitors and its knockout leads to severe brain malformations and to developmental arrests before birth [77, 78]. Interestingly, the relative abundance of genes involved in neuron differentiation and axon guidance has also been reported by two studies which compared the sperm methylome of fertile and infertile men [26, 79]. Because selection of the bulls in our study was based on the NRR 56, differences in developmental outcomes up to 56 days of gestation may contribute to subfertility. Gestational stages until 56 days are critical for neural tube patterning in cattle [80]; methylation changes affecting genes involved in this process may therefore compromise the viability of the embryo or fetus, hence affecting NRR 56.

Twenty-five differentially methylated genes were found in common between our study and four human studies [26–28, 52], and the same biological functions related to sperm physiology and development were affected in previous studies published on bull fertility [35–40]; thus strengthening the relevance of these genes and functions to male fertility. Another biological feature that appeared to be conserved in other studies conducted on fertility-related variations in the sperm methylome was that most differentially methylated loci were hypermethylated in subfertile or infertile cases when compared to controls in bulls [40], boars [81] and humans [28, 31, 52, 79, 82]. Because sperm cells are hypomethylated relative to somatic cells in many species, including humans [83] and cattle [43, 47], it has been proposed that the genome-wide erasure of DNA methylation was impaired during the differentiation of male germ cells in infertile men with spermatogenesis defects [82]. More recently, hypermethylation at DMRs has been attributed to a larger proportion of contaminating somatic cells in sperm samples from oligozoospermic patients [31]. However, these two hypotheses are unlikely to apply to the present study, since we demonstrated that hypermethylation at DMCs and DMRs in subfertile bulls was not due to residual somatic cells, and that subfertile bulls did not suffer from obvious spermatogenesis defects. The functional role of DNA methylation in gene expression cannot be predicted as a whole, because it varies as a function of the gene elements targeted by differential methylation [84]. Furthermore, many distant regulatory elements such as enhancers are still poorly characterized in cattle and will fall into the intergenic class, which is enriched among DMCs independently of their methylation status in subfertile bulls. The absence of significant transcriptional activity in sperm cells is a further complication encountered during functional analyses of the sperm methylome. Considering all these limitations, it can be speculated that the DNA methylation differences we detected in transcriptionally silent sperm cells may reflect suboptimal transcriptional activity, either earlier during a bull’s life for genes involved in sperm differentiation and physiology, or later after fertilization with respect to developmental genes, both processes resulting in subfertility.

In contrast with the overall hypermethylation of DMCs/DMRs in subfertile bulls, we observed a strong enrichment in LINE retrotransposons among hypomethylated DMCs, as well as a trend toward hypomethylation for LINEs in the L1 family but not the BovB family. Interestingly, the bovine genome contains more potentially active copies of L1 than of BovB retrotransposons; and consistent with this, L1 repeats also seem to have arisen more recently during evolution than the BovB family [85]. De novo DNA methylation guided to L1 repeats by PIWI-interacting RNAs (piRNAs) offers a safeguard against the mobilization of retrotransposons in the germline, and disruption of this defense mechanism in male mice leads to genome invasion and meiotic arrest [86], suggesting that the demethylation of L1 repeats might be at risk regarding the genome integrity of cattle germ cells. The hypomethylation of L1 has been reported in the testes of patients with spermatogenic failure, and associated with the down-regulation of genes in the piRNA pathway, suggesting that the molecular mechanisms involved in the epigenetic silencing of retrotransposons were not fully functional in these patients [87]. In line with this hypothesis, L1 expression has been claimed to increase in the testes of patients with impaired spermatogenesis [88]. However, other studies have not reported any significant changes to L1 DNA methylation in the sperm cells of infertile men [27, 30], which is also consistent with the small degree of difference we observed between fertile and infertile bulls. The expression of L1 repeats needs to be tightly regulated not only in germ cells but also after fertilization, where it regulates global chromatin accessibility in mouse embryos [89]; a loss of L1 silencing mechanisms in germ cells or embryos has also been proposed to cause early spontaneous miscarriage in humans [90]. Given the absence of severe spermatogenic defects in subfertile bulls, it is tempting to speculate that the slight hypomethylation we observed in sperm may lead to a suboptimal level or timing of the expression of L1 repeats after fertilization rather than to massive genome invasion during germ cell differentiation. This suboptimal expression may alter the dynamics of post-fertilization reprogramming, ultimately resulting in developmental arrest or pregnancy losses.

## Conclusion

Using the largest cattle cohort in the field and extensive DNA profiling analyses, we were able to demonstrate that the bull sperm methylome displays important inter-individual variability. This inter-individual variability remains poorly investigated in humans and deserves further attention, as it may reflect earlier exposures that could be passed to the next generation. We identified a facultative DNA methylation signature that predisposed to subfertility and from which fertility status for the whole career of the bulls could be predicted consistently in at least 70% of bulls from different breeding companies and of different ages. Our results are promising in terms of applications, but also suggest the existence of independent and/or compensatory mechanisms for the regulation of male fertility. Identifying these mechanisms will offer further opportunities regarding the practical use of these biomarkers to predict fertility.

## Methods

### Bull cohorts and semen samples

Semen samples from 120 French Montbéliarde bulls, grouped in two independent cohorts, were used for this study. All samples were prepared from frozen semen straws commercialized for AI and stored in liquid nitrogen.

The main cohort was used to generate DNA methylation data using RRBS, identify fertility-related DMCs and construct and validate the predictive model. This included 100 bulls born between 2011 and 2014 and commercialized by the breeding companies Umotest and Evajura, and maintained in two different semen collection centers located 100 km distant from each other in France (*n* = 56, center 1 and *n* = 44, center 2), 57 of which were classified as fertile and 43 as subfertile. Fertility was defined at the bull level based on the non-return rate at 56 days post-insemination (NRR 56), obtained as follows. For each bull, the AI outcomes were obtained from all the AIs performed using the semen of this bull in 2017, 2018 and 2021. Each AI was given a score of 0 if another AI of the mated cow was observed within the 56-day subsequent interval, and 1 otherwise. To eliminate any bias due to the spurious association with other factors, the bull fertility indicator was estimated with the linear model applied to the 0/1 score of all AIs in the population, and used in the French bovine genetic evaluation of female fertility for selection purpose [91]. This model included the fixed effects of herd-year, month-year, parity of the cow, interval between calving and insemination, week day, AI technician, category of semen (sexed vs. conventional), and the random effects of genetic and permanent environmental effects of the cow, and of the bull. The bull effect was assumed to be normally distributed with zero mean and variance equal to 0.01 phenotypic variance. The bull effect estimate was used in the present study and referred to as “corrected NRR 56”. To obtain one semen sample, 8–10 straws per bull that represented 2 to 5 ejaculates collected at 17–19 months of age and within a short period of time (6–52 days), were pooled after thawing for 30 s at 37 °C. Subsamples of 15 µL were used immediately to analyze semen functional parameters. The remaining semen was centrifuged for 7 min at 3500 g at room temperature and the extender was removed. Although microscopic examination did not reveal any significant contamination by somatic cells, the cell pellets were washed once with H_2_O. Unlike somatic cells, bull spermatozoa are resistant to this treatment, which therefore guaranteed the absence of any residual somatic cells in the samples. After a further wash in phosphate buffer saline (PBS 1×), the sperm pellets were resuspended in PBS 1× and divided into aliquots for various experimental purposes. The equivalent of one straw (20 million sperm cells) was used for DNA extraction.

The independent cohort was used to produce RRBS data and evaluate the potential of the model built on the main cohort for field applications. This included 20 bulls born between 2009 and 2012 and marketed by the breeding company Umotest. One ejaculate was analyzed per bull, whose age at semen collection ranged from 15 to 39 months. Ejaculates were defined as fertile (*n* = 16) or subfertile (*n* = 4) based on the corrected NRR 56 calculated for each bull and as explained above for the main cohort. One straw was used to assess semen functional parameters and extract DNA, as described above.

The entire process, from straw thawing to semen assessment and DNA extraction, was conducted in 7 batches of 2–24 samples per batch for the main cohort, and 7 batches of 1–6 samples per batch for the independent cohort. For each sample, information on the batch, the bull (year of birth, semen collection center), ejaculates (dates of collection, number of straws in the sample), the corrected NRR 56 and SCR are provided in Additional file 2: Table S1. Corrected SCR was obtained as described above for corrected NRR 56, except that each AI was given a score of 0 if no birth was reported for the mated cow after the expected gestation length, and 1 otherwise, as described by Barbat et al. [91].

### Semen functional parameters

Semen motility was assessed by computer-assisted semen analysis (CASA, IVOS II, Hamilton Thorne, IMV Technologies). Five µL out of 15 µL pooled semen were mixed with 10 µL Easy buffer B (IMV Technologies) and incubated for 5 min at 37 °C. After incubation, 4 µL of this mix was loaded into a standardized four-chamber counting slide (Leja), which was then placed into the CASA system. Sperm motility was averaged from 10 microscope fields analyzed using the predetermined starting point set within each chamber. Results were expressed as percentages of motile sperm in the sample. Sperm viability and mitochondrial status were assessed from 5000 cells using the easyCyte 8HT flow cytometer and CytoSoft software (Guava, IMV Technologies). For sperm viability, membrane integrity was assessed using EasyKit 1 Viability and Concentration (IMV Technologies) that stains viable spermatozoa with intact membranes in green. Results were expressed as percentages of viable sperm in the sample. Sperm mitochondrial status was assessed using the EasyKit 2 (IMV Technologies) and expressed as the percentage of polarized mitochondria that appeared in orange fluorescence. Both kits were used as described by Sellem et al. [92].

In order to account for variations between the different series of experiments, the data were corrected for the batch effect using a linear model, with the sample preparation batch (Additional file 2: Table S1) set as a fixed effect. Residuals of this model were extracted for further analyses. The results obtained for viability, mitochondrial status and motility, with and without correction for the batch effect, are provided in Additional file 3: Table S2 and Additional file 1: Fig. S8.

### Genomic DNA preparation

Approximately 20 million spermatozoa prepared as described above were pelleted and resuspended in 200 μL lysis buffer (10 mM Tris–HCl pH 7.5, 25 mM EDTA, 1% SDS, 75 mM NaCl, 50 mM dithiothreitol and 0.5 μg glycogen), and incubated overnight at 55 °C in the presence of 0.2 mg/ml proteinase K. After incubation with 25 μg/ml RNAse A for 1 h at 37 °C, genomic DNA was extracted twice using phenol:chloroform (1:1) and chloroform, then precipitated with ethanol and washed. The dried pellet was resuspended in TE buffer (10 mM Tris HCl pH 7.5, 2 mM EDTA) and the DNA concentration was measured using a Qubit 2.0 Fluorometer with the dsDNA BR Assay kit (Invitrogen). The integrity of genomic DNA was confirmed for all samples by agarose gel electrophoresis.

### Reduced representation bisulfite sequencing

RRBS libraries were produced in 12 batches for the main cohort and 4 batches for the independent cohort. All pipetting steps before final amplification were carried out using an NGS STARlet liquid handling system with four channels (Hamilton), ensuring reproducibility between the different library preparation batches. Genomic DNA (200 ng) was digested with MspI, end-repaired and ligated overnight with Illumina adapters [43, 93]. The following day, size selection was performed using SPRIselect magnetic beads (Beckman-Coulter) as previously reported [65]. The DNA was then converted twice with sodium bisulfite using the EpiTect bisulfite kit (Qiagen) following the manufacturer’s instructions. Converted DNA was amplified with Pfu Turbo Cx hotstart DNA polymerase (Agilent) using 14 PCR cycles. The libraries were purified using AMPure XP beads (Beckman-Coulter) and DNA concentrations were measured with a Qubit 2.0 Fluorometer with the dsDNA HS Assay kit (Invitrogen). Electrophoresis on a 4–20% precast polyacrylamide TBE gel (Invitrogen) and staining with SYBR green confirmed the homogeneous pattern for all libraries, with fragments ranging from 150 to 400 bp (40–290 bp genomic DNA fragments + adapters). The libraries were finally sequenced on an Illumina HiSeq4000 sequencer to produce 75 bp paired-end reads (Integragen SA).

### Bioinformatics analyses

On average, sequencing generated 33 and 35 million read pairs per library for the main cohort and independent cohort, respectively. The sequences displayed the expected nucleotide composition based on MspI digestion and bisulfite conversion according to FastQC quality control (https://www.bioinformatics.babraham.ac.uk/projects/fastqc/). Subsequent quality checks and trimming were carried out using TrimGalore v0.4.4 (https://www.bioinformatics.babraham.ac.uk/projects/trim_galore/), which removed adapter sequences, poor quality bases (Phred score below 20) and reads shorter than 20 nucleotides. The bisulfite conversion rate was estimated from unmethylated cytosine added in vitro during the end-repair step. This reached 99% on average (Table 1) and the minimal value for all 120 samples was 98.1%. High quality reads were aligned on the bovine reference genome (ARS-UCD2.1 assembly) using Bismark v0.20.0 in the default mode with Bowtie 1.2.1 [94, 95]. Only CpGs covered by at least 10 uniquely mapped reads (CpGs10) were retained for subsequent analysis. To avoid the confounding effects of sequence polymorphisms and bisulfite conversion, 401,172 CpGs10 that co-localized with a putative sequence polymorphism affecting the C and/or G were filtered out [96]. Since no sequence polymorphism information was available for unplaced scaffolds, CpGs located on unplaced scaffolds were also filtered out. Each remaining CpG10 was assigned a methylation percentage per sample calculated from Bismark methylation calling $\left( {{\text{number}}\;{\text{of}}\;{\text{reads}}\;{\text{with}}\;``C'' \times 100} \right)/\left( {{\text{number}}\;{\text{of}}\;{\text{reads}}\;{\text{with}}\;C + {\text{number}}\;{\text{of}}\;{\text{reads}}\;{\text{with}}\;``T''} \right)$, which could be visualized using the Integrative Genome Viewer (IGV) genome browser [97]. PCA and hierarchical clustering were then computed on the matrix of methylation percentages for each CpG10 with no missing values, using the FactoMineR R package [98]. For hierarchical clustering, the distance between samples was calculated using Pearson correlation coefficients and Ward’s method was applied as the linkage function.

A subset of 1,548,563 CpGs10, covered in at least 22 samples per fertility group of the main cohort, constituted the background. The DMCs were identified from this background using methylKit v1.0.0 [49] and DSS v2.14.0 [48] in the default mode. With methylKit, a CpG10 was considered as a DMC when the adjusted *p* value (*q* value, obtained after SLIM correction) was lower than 0.01 and the average methylation difference between the two groups was at least 10% according to the methylKit calculation mode, which takes account of the coverage per sample. With DSS, the same minimal methylation difference applied, but the threshold for the adjusted *p* value was set at 0.1, and *p* value adjustment was performed according to the Independent Hypothesis Weighting method, using the alpha parameter set at 5% and the average methylation per group as a covariable [99]. DMRs were defined as regions containing at least 3 DMCs with an inter-distance between each DMC of 100 bp or less. It should be noted that both the DMC and DMR datasets contained missing values because of samples in which the coverage thresholds were not reached.

For Fig. 2C, an average DNA methylation value per DMR and per sample was calculated from all the CpGs10 included in the DMR. The heatmap was then generated for 18 DMRs displaying DNA methylation values for all 100 samples using the R package pheatmap (v1.0.12).

Annotation of the background CpGs10, DMCs and DMRs was performed as described [65] relative to gene features, CpG density and repetitive elements using an in-house pipeline. The reference files were downloaded from Ensembl (ftp://ftp.ensembl.org/pub; release 95). The following criteria were applied: TSS, − 100 to + 100 bp relative to the transcription start site (TSS); promoter, − 2000 to − 100 bp relative to the TSS; TTS, − 100 to + 100 bp relative to the transcription termination site (TTS); shore, up to 2000 bp from a CGI, and shelf, up to 2000 bp from a shore. A site/region was considered to belong to a CGI (respective shore and shelf) if an overlap of at least 75% was observed between the site/fragment and the CGI (respective shore and shelf). A site/region was considered as being overlapped by a repetitive element whatever the extent of this overlapping. The lists of fertility-related DMCs and DMRs with annotation features are provided in Additional files 4 and 5: Tables S3 and S4, respectively.

Genes containing DMCs in intragenic regions and/or in the upstream (up to − 10 kb from the TSS) and downstream (up to + 10 kb from the TTS) regions were subjected to an enrichment analysis using DAVID with default parameters [100]. The 19,829 genes covered by RRBS (i.e., all genes containing at least one background CpG10) were used as the reference. Clusters with an enrichment score above 1.3 were taken into account.

To better characterize repetitive elements, an artificial genome containing the consensus sequence of each bovine repeat was constituted from the Repbase database [46]. Reads were aligned on this artificial genome as explained above, and the average methylation rate was calculated per CpG10 for each repeat and each sample. A differential methylation analysis between fertile and subfertile bulls was then performed using methylKit [49], as described above.

### Bisulfite pyrosequencing

For five genomic regions, ten samples per fertility group were selected based on their contrasting methylation patterns under RRBS analysis (Additional file 9: Table S8). Bisulfite conversion was performed on 0.5 µg genomic DNA using the EpiTect bisulfite kit (Qiagen). Primers were designed using the Qiagen Pyromark assay design software (Additional file 10: Table S9) and amplifications were performed using the Pyromark PCR kit (Qiagen) according to the manufacturer’s instructions. The following PCR program was used: 15 min at 95 °C followed by 45 cycles of 30 s at 94 °C, 30 s at 56 °C, 30 s at 72 °C, and finally 10 min at 72 °C. The reverse primers were 5′-biotinylated for all five regions. After denaturation and washes, the purified biotinylated strand of PCR products was employed as a template for pyrosequencing with 0.3 µM pyrosequencing primer, using the Pyromark Q24 device and Pyromark Gold Q96 reagents (Qiagen). Each CpG was analyzed in duplicate, and inconsistent duplicates (more than 5% difference) were repeated. For each sample, the DNA methylation percentage per CpG was obtained by calculating the mean of all consistent replicates that passed quality control by the Pyromark Q24 software. DNA methylation values obtained by pyrosequencing were compared between fertile and subfertile bulls using a Wilcoxon test. The significance of correlations between the average DNA methylation values obtained by RRBS and pyrosequencing for each region was tested using Spearman’s rank correlation test.

### Random Forest predictive model

The predictive model was built on the matrix of methylation percentages at DMCs, where samples were in rows and DMCs in columns. The DMCs with missing values were either filtered out before model construction or conserved if they contained less than 10% missing values; the latter were then imputed using the R package missMDA with default options [101]. Random Forest models were built using the ‘rf’ option from the R package caret (v6.0-84) [102]. The “mtry” parameter was estimated by the square root of the number of features in the model, and the number of trees was set at 500.

Three different strategies were applied to assess the performance of the predictive model (Fig. 7), and the predicted fertility status was compared with the actual fertility status of samples included in each type of testing set. For this purpose, ROC curves were computed [103], and four model quality indicators were calculated: model accuracy (correct prediction rate), AUC, sensitivity (true positive rate) and specificity (true negative rate). For the first strategy with 50 resamplings of the testing test, the average accuracy, AUC, sensitivity and specificity were considered to evaluate the model performance by cross-validation.

### Other statistical analyses

The groups were compared using the non-parametric Wilcoxon test if the following criteria did not apply: (1) more than 15 values available per group; (2) normal distribution according to the Shapiro–Wilk test; (3) same variance of the two groups according to the F-test. Among a series of comparisons, the Wilcoxon test was used for all comparisons regarding consistency, even if some comparisons fulfilled the above criteria.

### Literature mining

A systematic review of the literature was performed for all genes differentially methylated with the Biomart annotation available (“Gene name” column in Additional file 4: Table S3; 139 genes out of 170). The NCBI Pubmed database was interrogated using the gene names successively associated with each of the following terms: “embryo*”, “sperm*” and “fertility”, and all the relevant references were collected (Additional file 7: Table S6).

The comparison with human case studies was performed as follows: among all the listed references, those focusing on the comparison of genome-wide DNA methylation patterns between fertile and infertile/subfertile human sperm samples, and with accessible supplementary data available, were pointed out. This led to the selection of four studies highlighting 1843 [27], 31 [26], 3 [28] and 384 [52] differentially methylated genes. These four gene lists were compared with the 139 genes with the Biomart annotation available identified during the current study. The genes found in common are listed in Additional file 8: Table S7. For studies where the information was available, the methylation status in subfertile samples is indicated.

## Supplementary Information

**Additional file 1: Fig. S1.**. correlation clustering run on the methylation percentages at CpGs10 covered in at least 22 samples per group. **Fig. S2**: heatmap run on CpGs discriminant between sperm and somatic cells. **Fig. S3**: annotation of fertility-related DMRs relative to different genome features. **Fig. S4**: average DNA methylation at individual LINE and LTR repeats after alignment of the RRBS sequences on a Repbase artificial genome. **Fig. S5**: pyrosequencing validation of the upstream region of LBX1 gene. **Fig. S6**: fertility, semen functional parameters and semen sample characteristics in correctly classified and misclassified bulls. **Fig. S7**: PCA run on the methylation percentages at DMCs, with or without the imputation of missing values. **Fig. S8**: semen functional parameters before and after correction for the batch effect.

**Additional file 2: Table S1.** Listing the semen samples, information on their origin and processing, and the field fertility of the corresponding bulls.

**Additional file 3: Table S2.** Listing the functional parameters measured on each semen sample.

**Additional file 4: Table S3.** Listing the fertility-related DMCs, their DNA methylation status in each sample and their annotation regarding genome features.

**Additional file 5: Table S4.** Listing the fertility-related DMRs, their methylation status in each fertility group and their annotation regarding genome features.

**Additional file 6: Table S5.** Listing the CpGs10 obtained after alignment of the RRBS sequences on a Repbase artificial genome, their coverage and DNA methylation status in each sample.

**Additional file 7: Table S6.** Listing the differentially methylated genes relevant to fertility together with supporting references.

**Additional file 8: Table S7.** Listing the differentially methylated genes found in common with four human studies.

**Additional file 9: Table S8.** Listing the semen samples used for pyrosequencing validation.

**Additional file 10: Table S9.** Listing the primers used for pyrosequencing validation.

## Data Availability

Additional data files are provided (see above). RRBS fastq files have been deposited in the European Nucleotide Archive (ENA) at EMBL-EBI under accession number PRJEB46371 (https://www.ebi.ac.uk/ena/data/view/PRJEB46371).

## Abbreviations

AI: Artificial insemination
AUC: Area under the ROC curve
CASA: Computer-assisted semen analysis
CGI: CpG Island
DAVID: Database for annotation, visualization and integrated discovery
DMC: Differentially methylated CpG
DMR: Differentially methylated region
IGV: Integrative genome viewer
NRR 56: Non-return rates of inseminated cows at 56 days post AI
PBS: Phosphate buffer saline
PCA: Principal component analysis
ROC: Receiver operating characteristics
RRBS: Reduced representation bisulfite sequencing
SCR: Sire conception rate
TSS: Transcription start site
TTS: Transcription termination site
UTR: Untranslated region
