# Supplementary material for: Predicting male fertility from the sperm methylome: application to 120 bulls with hundreds of artificial insemination records
Source: Clin Epigenetics. 2022 Apr 27;14:54. doi: 10.1186/s13148-022-01275-x (PMC9047354; doi:10.1186/s13148-022-01275-x)
Supplement: Supplementary file 1 — Additional file 1: Fig. S1.. correlation clustering run on the methylation percentages at CpGs10 covered in at least 22 samples per group. Fig. S2: heatmap run on CpGs discriminant between sperm and somatic cells. Fig. S3: annotation of fertility-related DMRs relative to different genome features. Fig. S4: average DNA methylation at individual LINE and LTR repeats after alignment of the RRBS sequences on a Repbase artificial genome. Fig. S5: pyrosequencing validation of the upstream region of LBX1 gene. Fig. S6: fertility, semen functional parameters and semen sample characteristics in correctly classified and misclassified bulls. Fig. S7: PCA run on the methylation percentages at DMCs, with or without the imputation of missing values. Fig. S8: semen functional parameters before and after correction for the batch effect. [file 13148_2022_1275_MOESM1_ESM.pdf]

ADDITIONAL DATA FILE

Predicting male fertility from the sperm methylome: application to 120 bulls with hundreds of artificial insemination records

Valentin Costes[1,2,3,4], Aurélie Chaulot-Talmon[1,2], Eli Sellem[1,2,3], Jean-Philippe Perrier[1,2], Anne Aubert-Frambourg[1,2], Luc Jouneau[1,2], Charline Pontlevoy[1,2], Chris Hozé[3,4], Sébastien Fritz[3,4], Mekki Boussaha[4], Chrystelle Le Danvic[3], Marie-Pierre Sanchez[4], Didier Boichard[4], Laurent Schibler[3], Hélène Jammes[1,2], Florence Jaffrézic[4], Hélène Kiefer[1,2]*

[1]Université Paris-Saclay, UVSQ, INRAE, BREED, 78350 Jouy-en-Josas, France.

[2]Ecole Nationale Vétérinaire d'Alfort, BREED, 94700, Maisons-Alfort, France

[3]R&D Department, ALLICE, 149 rue de Bercy, 75012, Paris, France.

[4]Université Paris-Saclay, AgroParisTech, INRAE, GABI, 78350 Jouy-en-Josas, France.

*Corresponding author: helene.kiefer@inrae.fr

Additional results: a larger panel of DMCs with imputed DNA methylation values did not enhance the performance of the predictive model

Because of both the large size of the main cohort and the bioinformatics settings, most of the fertility-related DMCs contained missing values (383 out of 490) and were therefore not used to build the model. To verify whether methylation at these DMCs contained additional information that could be used to improve the model, DNA methylation values were imputed to an extended panel of 295 DMCs containing no more than 10% missing values. Principal component analysis run on DMCs without and with imputation did not reveal any significant changes to the percentage of explained variance or in individual factor maps (Supplementary Figure 7). The performance of the new model was also very similar to that obtained using the 107 DMCs without missing values (AUC: 0.83, accuracy: 0.76, sensitivity: 0.83 and specificity: 0.59; averaged values after 50 resamplings of the training and testing sets). These results therefore indicate that DNA methylation at both panels of fertility-related DMCs enabled the prediction of some, but not all, cases of subfertility. The similar results obtained using 107 DMCs with no missing values and 295 DMCs with the imputation of missing values suggest either a certain degree of redundancy among DMCs for the prediction of fertility, or that imputation is not sufficiently precise to provide any additional information. In light of these results, we therefore focused on the model without imputed values.

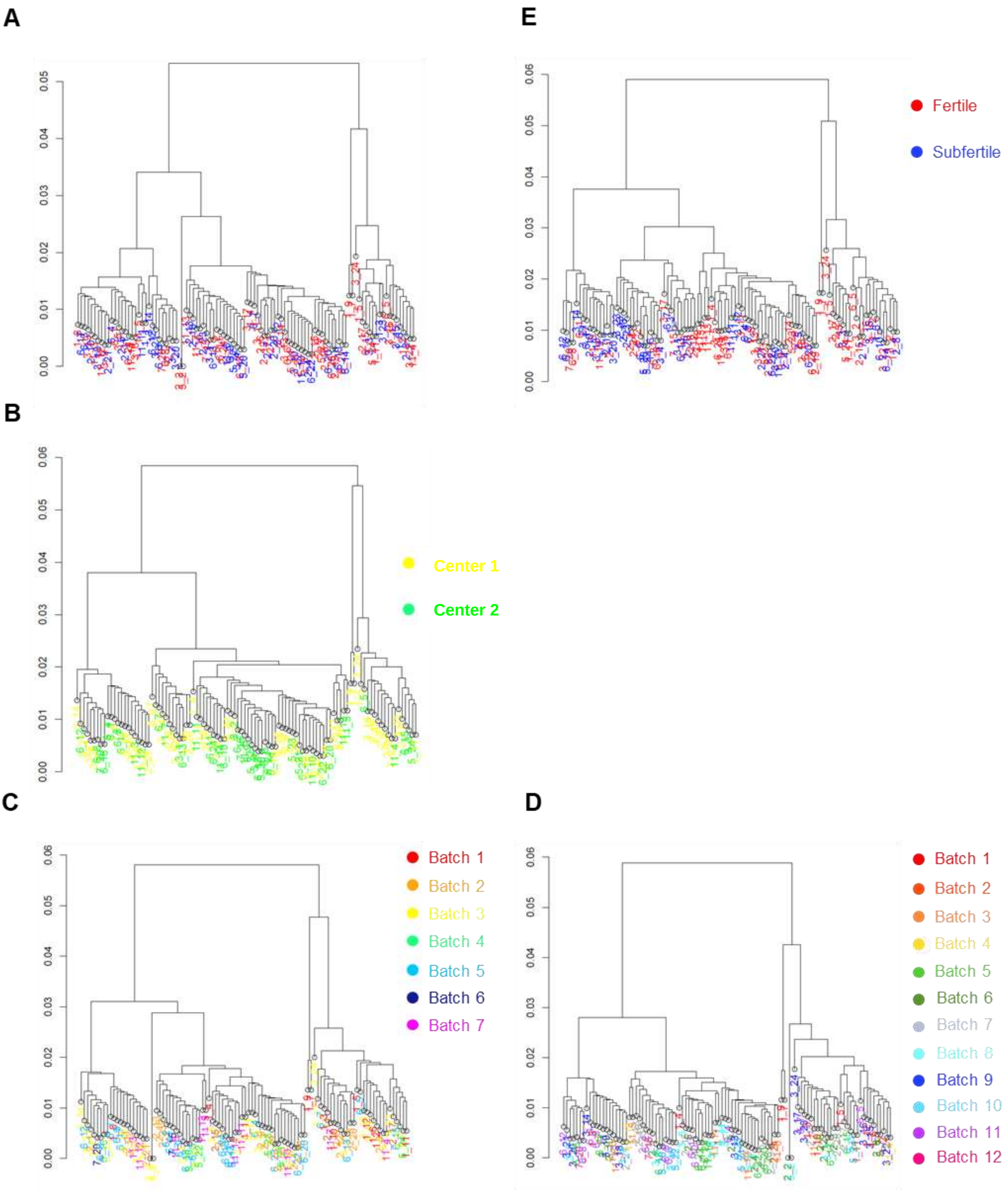

Supplementary Figure 1. Correlation clustering was run on the methylation percentages calculated at CpGs covered by at least 10 reads in at least 22 samples per group in the main cohort, without (**A-D**) or with (**E**) putative sequence variants. **A, E:** samples from the fertile and subfertile bulls are shown in red and blue, respectively. **B:** samples originating from two semen collection centers are shown in yellow and green, respectively. **C:** samples obtained from different semen processing batches are displayed in different colors. **D:** samples obtained from different RRBS library preparation batches are displayed in different colors. Taken together, the results demonstrate that inter-individual variability unrelated to fertility shapes DNA methylation patterns, which are unaffected by confounding effects such as the origins of bulls or technical issues.

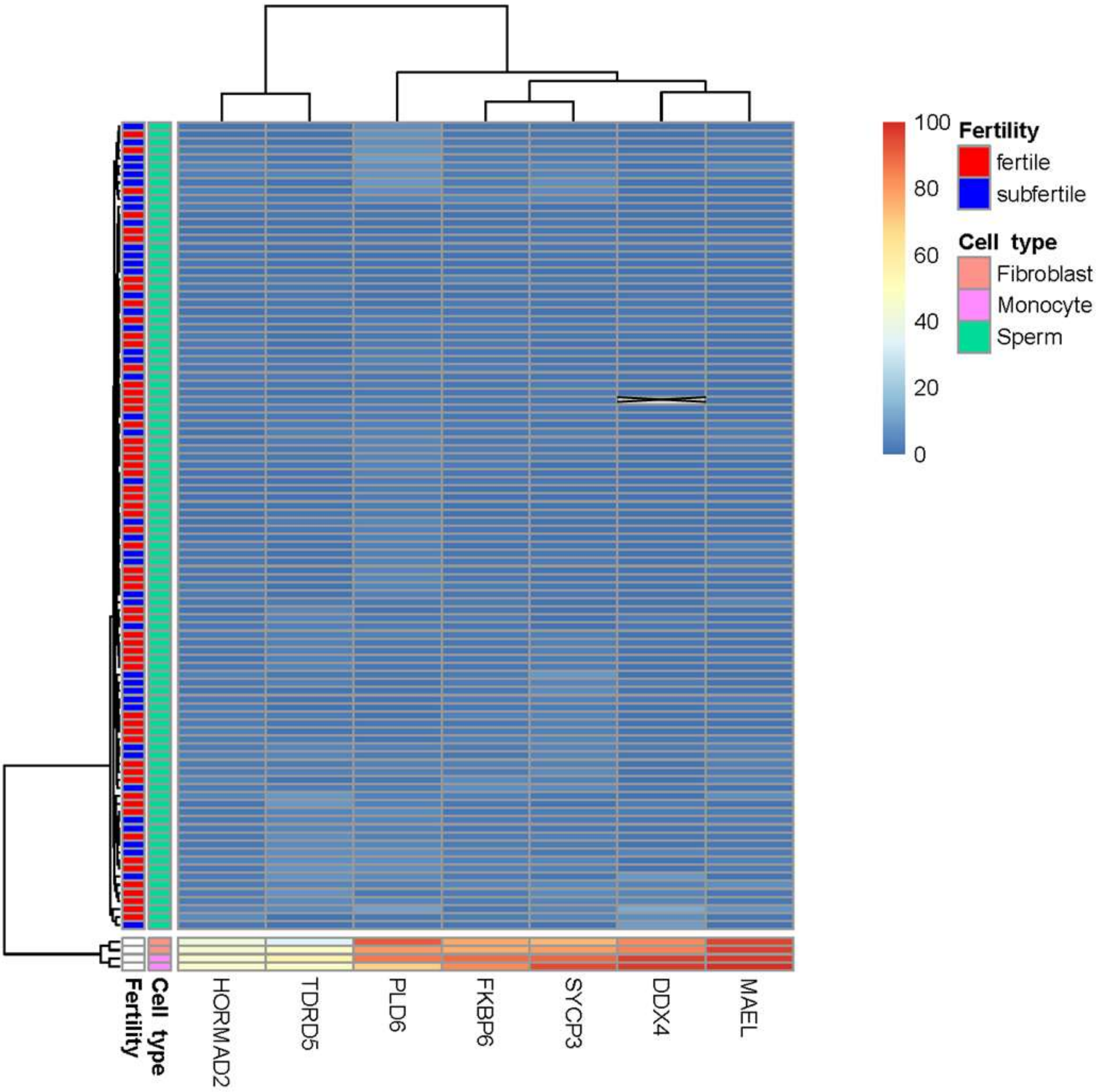

Supplementary Figure 2. Heatmap run on the 100 semen samples in the main cohort and on two types of adult somatic cells (monocytes and fibroblasts). Average DNA methylation values from promoters of genes involved in male gamete generation and at which DNA methylation enables the discrimination of sperm and somatic cells (Perrier et al., 2018) were used to build the heatmap. The black cross indicates that one semen sample was not covered at *DDX4*. Reduced representation bisulfite sequencing data for somatic cells are available under accession GSE102169. The absence of DNA methylation in any of the 100 semen samples confirms that they are not contaminated by somatic cells. Moreover, the 100 semen samples are not clustered according to fertility, demonstrating that residual somatic contamination, if any, does not confound the DNA methylation results related to fertility.

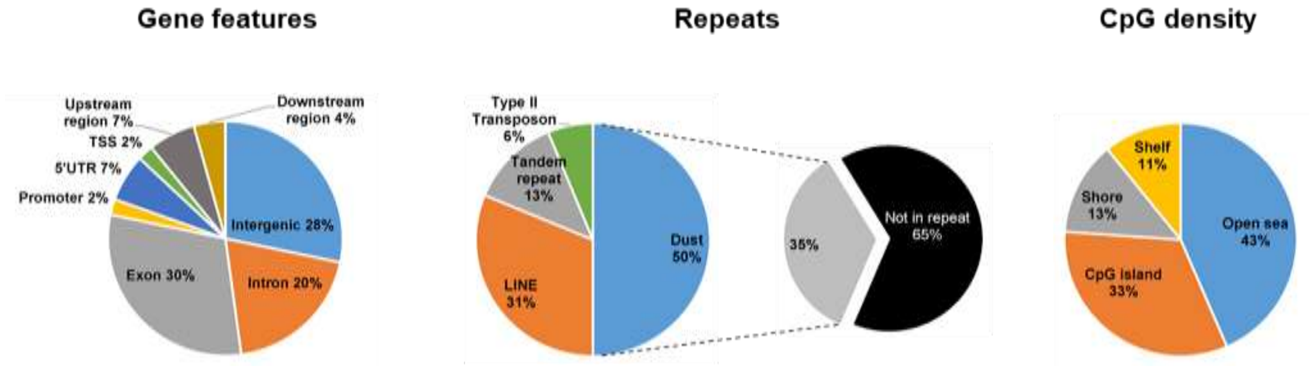

Supplementary Figure 3. The regions differentially methylated between fertile and subfertile bulls were annotated relative to gene features, repeats, and CpG islands, shores and shelves. Consistent with the fact that most of them were hypermethylated in subfertile bulls, the genome features targeted by DMRs are similar to those targeted by the DMCs that were hypermethylated in subfertile bulls (Figure 4A). TSS: transcription start site, UTR: untranslated region, upstream region: from -10 to 0 kb relative to the TSS; downstream region: from 0 to +10 kb relative to the transcription termination site.

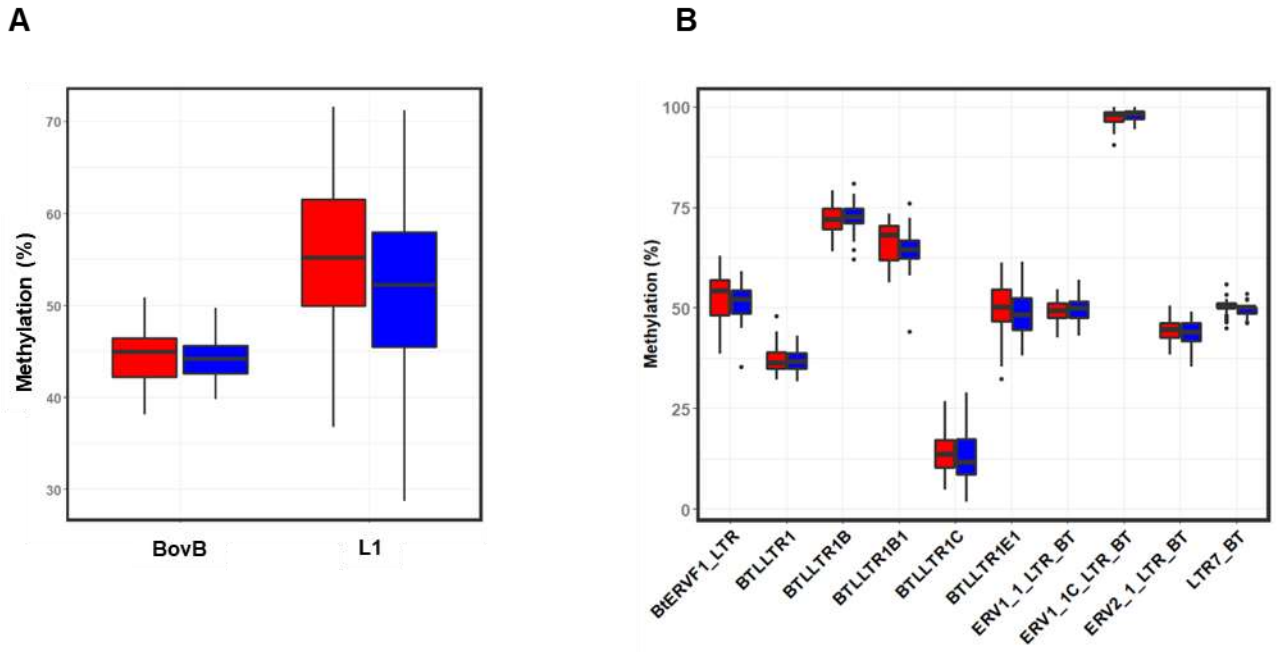

Supplementary Figure 4. RRBS sequences were aligned on a Repbase artificial genome and the average DNA methylation per individual repeat was calculated from the CpGs10 covered in each sample (Supplementary Table 5). Fertility groups were then compared using a Wilcoxon test. **A:** while DNA methylation was identical between fertile and subfertile bulls at LINE BovB, a tendency towards a slight decrease was observed in subfertile bulls at LINE L1 (Wilcoxon test, pvalue=0.072). **B:** the same approach was applied to members of the LTR family, but no difference could be found between fertile and subfertile bulls.

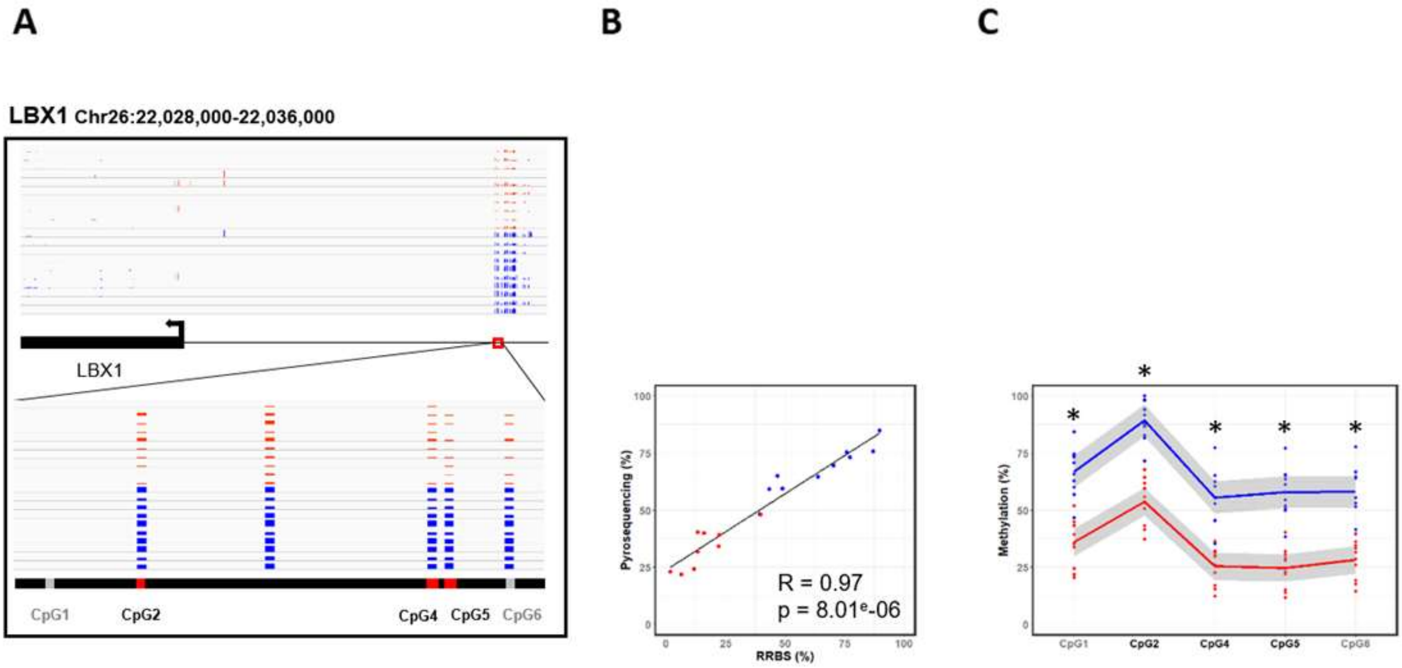

Supplementary Figure 5. A: IGV browser view of the upstream region of the *LBX1* gene. The zoomed view (lower panel) indicates the region targeted by pyrosequencing. The red and blue bar charts represent the methylation percentages at each CpG10 position for fertile (n=10) and subfertile (n=10) bulls, respectively. The CpGs analyzed by pyrosequencing are numbered according to their 5'-3' position along the genome. The CpGs identified as fertility-related DMCs are indicated in black text and red boxes, while non-DMCs are indicated in grey. **B:** The average methylation percentage measured by pyrosequencing (y-axis) was calculated for the three DMCs and plotted against the average methylation percentage measured by RRBS (x-axis). Each dot represents one semen sample from the fertile (in red, n=9) and subfertile (in blue, n=10) groups. The least squares line of best fit and Spearman's R rank correlation coefficient are indicated. **C:** Methylation percentages of individual CpGs assayed by pyrosequencing in fertile (in red, n=10) and subfertile (in blue, n=10) bulls. CpGs are numbered according to **A** and DMCs are highlighted in black. Dots show the methylation levels of individual samples, while the trends per fertility group are indicated by red and blue lines. Asterisks indicate that the methylation percentage measured by pyrosequencing differs significantly between fertility groups for all analyzed CpGs (Wilcoxon test, $p<0.05$).

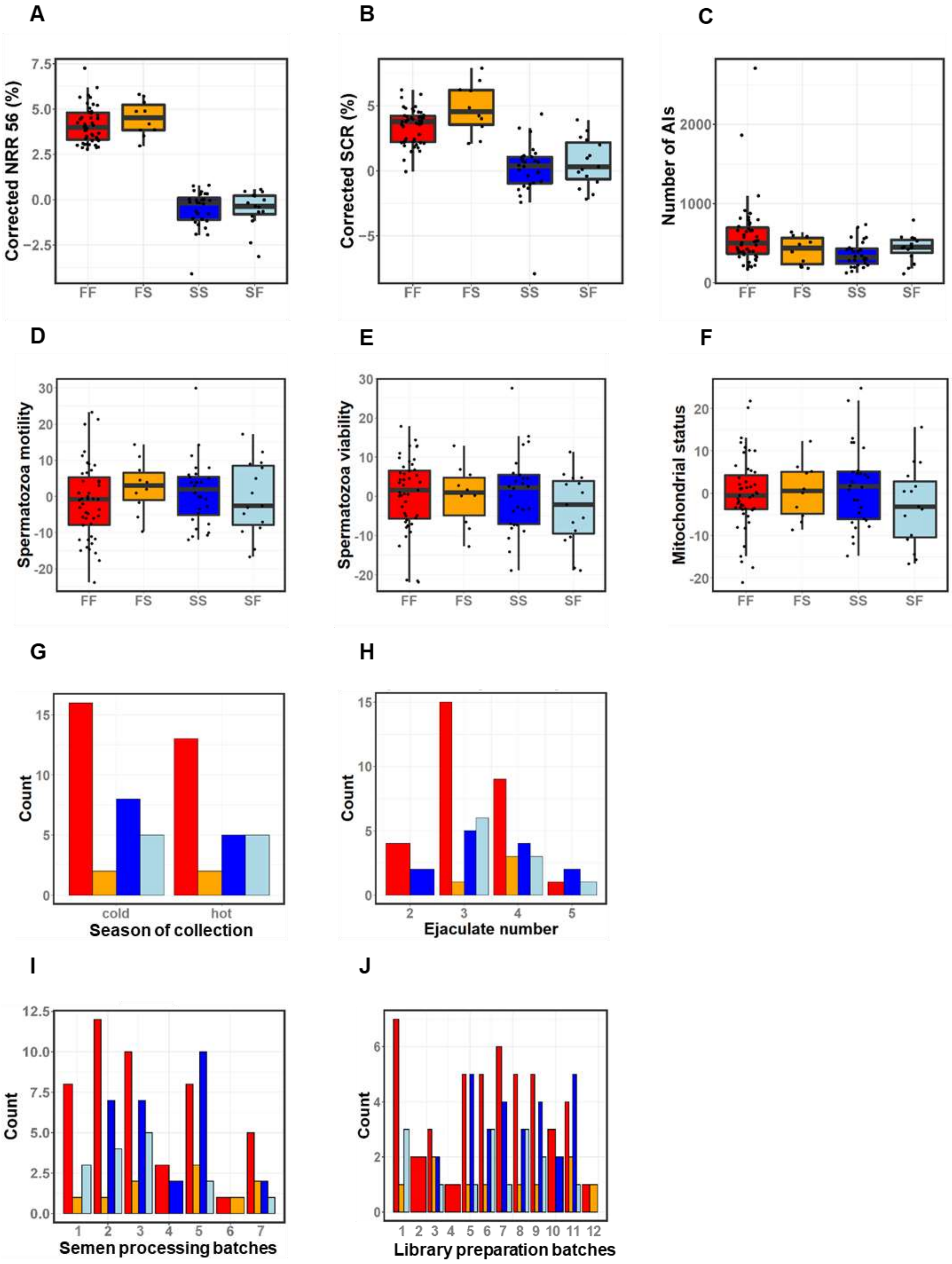
A
Corrected NRR 56 (%)
7.5
5.0
2.5
0.0
-2.5
FF
FS
SS
SF
B
Corrected SCR (%)
5
0
-5
C
Number of AIs
2000
1000
0
D
Spermatozoa motility
30
20
10
0
-10
-20
E
Spermatozoa viability
F
Mitochondrial status
G
Count
15
10
5
0
cold
hot
Season of collection
H
2
3
4
5
Ejaculate number
I
12.5
10.0
7.5
5.0
2.5
0.0
1
2
3
4
5
6
7
Semen processing batches
J
6
4
2
0
8
9
10
11
12
Library preparation batches

Supplementary Figure 6 (previous page). Within main cohort, correctly predicted bulls (FF: fertile predicted as fertile, in red; SS: subfertile predicted as subfertile, in blue) and misclassified bulls (FS: fertile predicted as subfertile, in orange; SF: subfertile predicted as fertile, in light blue) were compared for field fertility (**A-B**; **A:** corrected NRR 56; non return rate of the cows at 56 days post-insemination; **B:** corrected SCR; sire conception rate), the number of artificial inseminations used to evaluate fertility (**C**); and semen functional parameters (**D-F**; **D:** motility; **E:** viability; **F:** mitochondrial status). No significant differences between misclassified and correctly classified bulls from both fertility classes were observed (Wilcoxon test, adjusted pvalue>0.05). **G-J:** no obvious bias could be observed in the distribution of misclassified and correctly classified bulls regarding the season of semen collection (**G;** cold: from November to April; hot: from May to October; established according to temperature measurements during the years of collection), the number of ejaculates representing each sample (**H**), semen processing batches (**I**) and library preparation batches (**J**), demonstrating the absence of confounding factors in the experimental design.

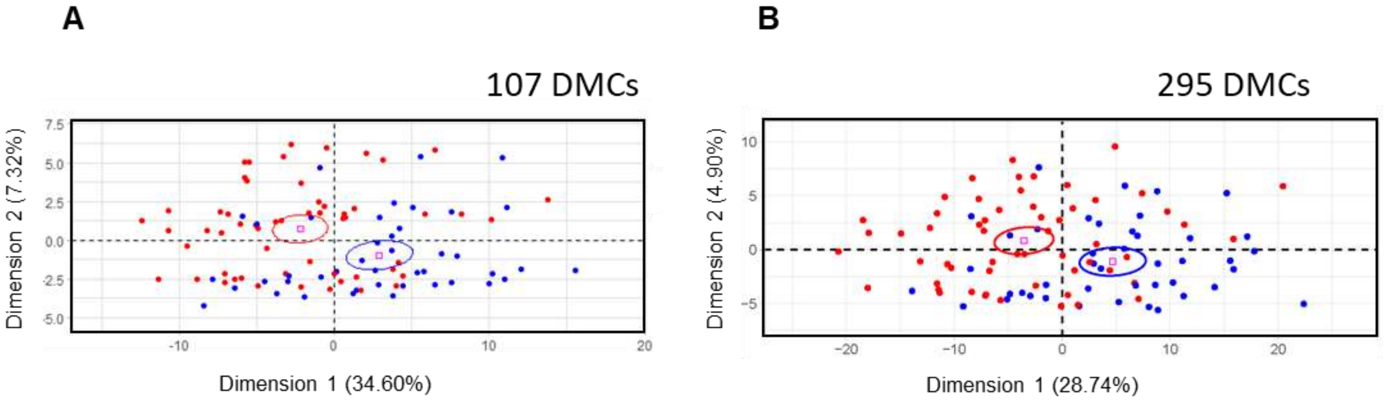

Supplementary Figure 7. Principal component analysis run on the differentially methylated CpGs identified between fertile (red) and subfertile (blue) bulls belonging to the main cohort. Confidence ellipses are represented. **A:** PCA run on 107 DMCs without missing values. **B:** PCA run on 295 DMCs after imputation of DNA methylation values at DMCs containing no more than 10% missing values. The results were similar in terms of the segregation of fertility groups and the percentages of variance explained by the first two dimensions.

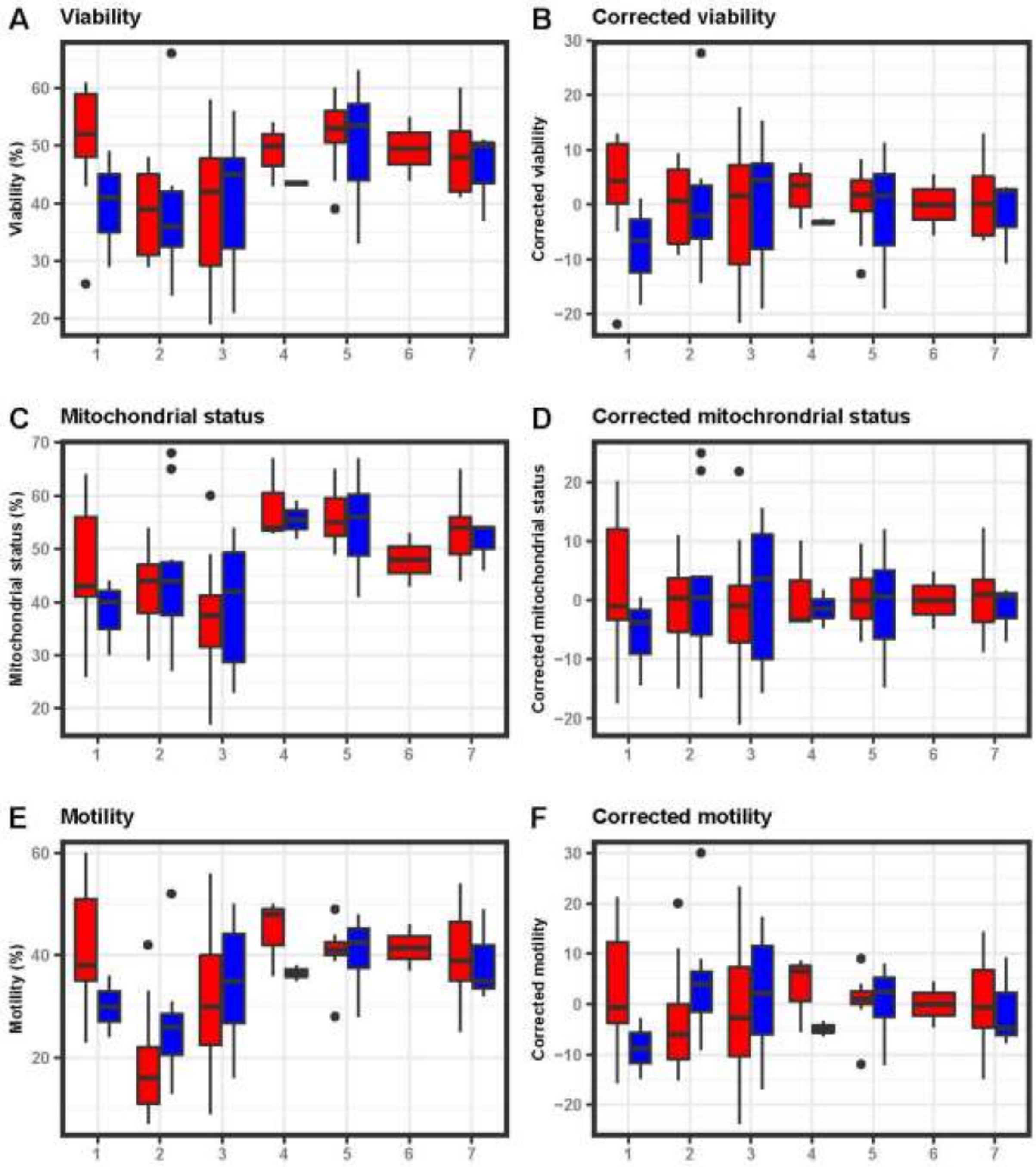

Supplementary Figure 8. Semen functional parameters measured on fertile (red) and subfertile (blue) semen samples before (**A, C, E**) and after correction for the batch effect (**B, D, F**). Due to the large size of the main cohort, semen samples were thawed and analyzed in seven batches of 2-24 samples. The seven batches are shown on the x-axis. The batch effect was no longer visible after correction, making it possible to analyze together the 100 samples whatever the batch during which they have been assayed.
